# Evaluation of integration in WHO’s tuberculosis, HIV, and antimicrobial resistance policies through the social-ecological lens

**DOI:** 10.1186/s12992-025-01150-3

**Published:** 2025-09-29

**Authors:** Jian Yang, Jiabin Xu, Christoph Benn, Xiaoyi Yu, Ying Chen, Shuduo Zhou, Zhongfei Pei, Yunxuan Hu, Ming Xu

**Affiliations:** 1 Department of Global Health, School of Public Health, Peking University, Beijing, China; 2 Institute for Global Health and Development, Peking University, Beijing, China; 3 Center for Global Health Diplomacy, Joep Lange Institute, Chemin du Pommier 42, Grand-Saconnex, 1218 Switzerland; 4 Department of Global Health, Peking University School of Public Health, 38 Xue Yuan Road, Haidian District, Beijing, 100191 China

**Keywords:** Tuberculosis, HIV, Antimicrobial resistance, Integration, World Health Organization, Policy analysis

## Abstract

**Background:**

TB, HIV, and AMR are closely related global health challenges. In the context of limited global health funds and insufficient resources, an integrated tuberculosis, HIV and antimicrobial resistance prevention and control method will play an important role in the optimization of resources and cost-effectiveness.

**Objective:**

This study aims to analyze the degree of policy integration for issues of tuberculosis, HIV and antimicrobial resistance in global health strategies and make recommendations for improving global health governance on related issues.

**Methods:**

We conducted a thorough analysis of global health policy documents from January 2015 to February 2024, using both quantitative and qualitative approaches. Our focus was on assessing the integration effectiveness of current global health governance mechanisms in addressing tuberculosis, HIV, and antimicrobial resistance from the global governance view based on the content analysis through word frequency analysis and thematic framework analysis. Besides, we conduct a thematic framework analysis of the action plans and policy recommendations outlined in the most recent reports from UNAIDS, Stop TB, and UNEP on HIV, TB and AMR.

**Results:**

The analysis revealed that most documents address TB, HIV, and AMR in isolation, with limited integration and intersectionality. TB and HIV are more frequently linked, while AMR is less associated with the other two. The proposed action lacks specific provisions for joint implementation or monitoring of the evaluation. Additionally, no documented comprehensive overview includes the overall framework of three health priorities.

**Conclusions:**

The study found that the current global health governance mechanism is significantly inadequate in dealing with integration solutions among tuberculosis, HIV and antimicrobial resistance. So we propose establishing integrated governance and coordination mechanisms for the same population at both horizontal and vertical levels, including individual, interpersonal, community, institutional, and societal levels, and developing an integrated policy framework to facilitate better resolution to address the association between TB, HIV infection and antimicrobial resistance in a resource-limited context.

**Clinical trial number:**

Not applicable.

**Supplementary information:**

The online version contains supplementary material available at 10.1186/s12992-025-01150-3.

## Abstract (significance)

What is already known about this topic

What this study adds

How this study might influence research, policy making and practice

There is a well-documented syndemic relationship between TB and HIV. The rise of antimicrobial resistance complicates the treatment of both TB and HIV. Multi-drug-resistant TB (MDR-TB) and extensively drug-resistant TB present significant challenges. Similarly, treatment-resistant strains of HIV are emerging, making integrated and effective treatment strategies crucial. How to seek an integrated approach to addressing tuberculosis, HIV and AMR has not been thoroughly explored, which can be critical to ending HIV/AIDS, tuberculosis and mitigating the threat of AMR given a lack of funding and limited resources worldwide.

This paper first evaluates the degree of integration in WHO’s Tuberculosis, HIV, and Antimicrobial Resistance Policies. Our analysis shows that most documents address TB, HIV, and AMR in isolation, with limited integration and intersectionality. The WHO and Stop TB, UNAIDS and UNEP have demonstrated varying degrees of integration in their action plans, governance and accountability mechanisms for TB, HIV and AMR with a high room for integration of policies from these institutions. The proposed action lacks specific frameworks, guidelines and regulations for joint implementation or monitoring of the evaluation.

An integrated evaluation of WHO’s tuberculosis, HIV, and AMR policies and analysis framework based on a Social-Ecological theory can help policymakers and researchers to identify specific policy gaps and raise encompassing recommendations with key determining actors for better addressing tuberculosis, HIV, and AMR in the context of limited financial resources.

**Supplementary information:**

The online version contains supplementary material available at 10.1186/s12992-025-01150-3.

## Introduction

Antimicrobial resistance poses a serious threat to the foundation of modern medicine and undermines the ability to maintain an effective global public health response to the persistent danger of infectious diseases. Tuberculosis (TB) is a major contributor to antimicrobial resistance, while Human Immunodeficiency Virus (HIV) drug resistance can lead to increased HIV infections and HIV-associated morbidity and mortality [1]. It is estimated that bacterial antimicrobial resistance (AMR) was directly responsible for 1.27 million global deaths in 2019 and contributed to 4.95 million deaths [2]. The World Bank estimates that AMR could result in US$1 trillion in additional healthcare costs by 2050, and US$ 1 trillion to US$ 3.4 trillion gross domestic product (GDP) losses per year by 2030 [3]. On the other hand, drug-resistant tuberculosis accounts for one-third of AMR globally, and HIV drug resistance (HIVDR) is gradually becoming an increasingly big health adjustment [1, 4–6].

Tuberculosis (TB) is the number one cause of death among people living with HIV in Africa, and a leading cause of death among people living with HIV worldwide [5, 7]. A total of 1.3 million people died from TB in 2022 (including 167 000 people with HIV)， only about 2 in 5 people with drug resistant TB accessed treatment, and 54% of TB patients known to be living with HIV were on antiretroviral therapy in 2022 [1, 8]. There were an estimated 39.0 million [33.1–45.7 million] people living with HIV at the end of 2022 with 86% [73– > 98%] knowing their status, 76% [65–89%] receiving antiretroviral therapy and 71% [60–83%] having a suppressed viral loads [4, 5]. Therefore, co-morbidity is the biggest cause of tuberculosis death, and tuberculosis is one of the leading causes of acquired immune deficiency syndrome(AIDS) death. Thus, people with microbial resistance, tuberculosis and AIDS are closely related and are one of the most severe global health threats.

Ending the TB epidemic and HIV epidemic by 2030 is among the health targets of the United Nations Sustainable Development Goals (SDGs) and addressing microbial drug resistance is one of the major health challenges in global health in recent and future decades. However, in many high TB burden countries, many proven approaches and tools have been applied inconsistently or without a systematic approach, mainly due to a lack of resources and coordination, which led to limited gains and missed opportunities to halt transmission [9]. Microbial drug resistance also faces the problems of insufficient funds and resources, tracking Antimicrobial Resistance Self-Assessment Survey (TrACSS) 2023 data show only 27% of Member States monitored and implemented their national action plans effectively and only 11% of Member States had allocated national budgets for implementation [10, 11]. There are also many funding gaps for HIV control [12]. The emergence and spread of AMR, tuberculosis and HIV have resulted in significant adverse impacts on public health, clinical, and economic outcomes [13].

The World Health Organization (WHO) governing body plays a key role in developing global health policy and guiding Member States’ action. Therefore, it is necessary for WHO to develop more effective strategies and action plans to address tuberculosis, HIV and drug resistance in the context of resource constraints. In the relevant guideline recommendations, the integrated prevention and control of tuberculosis and HIV is currently recommended, and the importance of HIV resistance and TB resistance is also mentioned in the prevention and control of AMR [9–11]. Many documents propose to promote the integration of HIV, tuberculosis and AMR at the national level, while relatively few guidelines for highly prevalent infectious diseases are considering resistance at a local level [14–17]. Besides, tuberculosis is not sufficiently emphasized in the AMR discussion at the highest decision-making level [18]. To date, the strategies and directives aimed at curbing antimicrobial resistance (AMR) within the healthcare sector are predicated on data furnished by clinical laboratories and monitoring mechanisms, which often employ comparable metrics but utilize assessment tools that lack standardization [19]. The concerns surrounding the accuracy, dependability, and consistency of such data have been largely overlooked.

### The role of WHO governance and the rationale for integration

The World Health Organization (WHO) governing bodies—namely the World Health Assembly (WHA) and the Executive Board (EB)—play a pivotal role in global health governance. WHA resolutions and EB decisions function as normative instruments that establish global health priorities, set standards, and guide Member States’ policies and actions [20]. Although not legally binding, these instruments carry significant political weight and provide a framework for national strategic planning, resource mobilization, and international cooperation [21]. They signal global consensus and guide the technical work of the WHO Secretariat, regional offices, and partner organizations.While the WHOs policy documents are influential in setting global priorities, the application of these policies at the country level remains contingent upon national health systems and their capacity to integrate or adapt these frameworks. The analysis of WHOs TB, HIV, and AMR resolutions highlights the potential for integration; however, the actual adoption and adaptation of these documents vary by country, influenced by local epidemiological data, resource availability, and national health priorities.

A critical question arises as to why service integration should be pursued at the WHO policy level when implementation ultimately occurs nationally. While countries constitute the primary implementing entities responsible for integrating health services according to their specific epidemiological and systemic contexts, integrated global guidance from WHO remains indispensable for several reasons. Firstly, integrated WHO policies can dismantle global-level silos that often mirror national-level fragmentation, particularly concerning funding streams and programmatic structures. Donors and international partners frequently align their support with WHO’s strategic priorities; consequently, fragmented guidance perpetuates fragmented funding and implementation [22]. Secondly, WHO’s integrated frameworks provide countries with essential technical tools, benchmarks, and best-practice models to effectively design and implement integrated programs. For instance, WHO’s guidance on collaborative TB/HIV activities has been instrumental in scaling up integrated services globally [23].

Furthermore, many low- and middle-income countries (LMICs) rely extensively on WHO’s technical guidance to formulate national strategic plans [24]. When WHO presents tuberculosis (TB), human immunodeficiency virus (HIV), and antimicrobial resistance (AMR) as interconnected challenges within a unified framework, it enables countries to adopt a holistic approach rather than addressing them as separate, competing priorities. Successful national-level integration frequently builds upon the foundation established by global normative guidance. Empirical evidence from Ghana demonstrates that the introduction of integrated TB/HIV services, guided by WHO policies, yielded significant improvements in treatment success rates [25]. Similarly, implementation experiences in South Africa and Uganda illustrate both the benefits and the operational challenges of integrated care delivery, underscoring the necessity for robust global frameworks to support national efforts [22, 26]. Therefore, analyzing the degree of integration within WHO’s highest-level policy documents serves as a critical proxy for assessing the global commitment to a synergistic response to these interconnected epidemics.

### Why an integrated approach is entailed?

In 2015, at the 67th World Health Assembly, World Health Organization (WHO) released a Global Action Plan on Antimicrobial Resistance—the blueprint for tackling AMR, in coordination with the Food and Agriculture Organization of the United Nations (FAO) and the World Organisation for Animal Health (OIE) [27]. Consecutively, in September 2016, at the United Nation General Assembly (UNGA), global leaders, for the first time, committed to act on antimicrobial resistance. Heads of State pledged to adopt a comprehensive, coordinated strategy and national plans to tackle the underlying causes of antimicrobial resistance (AMR) across various sectors, particularly in human health, animal health, and agriculture [28]. In July 2017, the G20 leaders recognized that action on tuberculosis as part of global efforts to confront antimicrobial resistance is critical [29]. During the 144th session of the Executive Board, convened in January 2019, the Director-General of the World Health Organization (WHO) articulated a commitment to fostering a cohesive and effective synergy among the various actions undertaken by the organization [30]. This strategic integration is intended to align the follow-up actions from the high-level meeting with the resolutions emanating from other closely related high-level meetings convened by the United Nations General Assembly. The Director-General emphasized the importance of this harmonization, particularly in relation to critical global health issues such as human immunodeficiency virus and acquired immunodeficiency syndrome (HIV/AIDS), antimicrobial resistance, and noncommunicable diseases (NCDs). At the 154th convening of the Executive Board in January 2024, the EB154/13 report underscored the imperative for a comprehensive and intersectoral public health strategy to combat antimicrobial resistance. It highlighted the critical interconnections between the priorities set forth and the resolutions of the Health Assembly, as well as the alignment with overarching global strategies and initiatives, particularly in the domains of tuberculosis and HIV management. Most recently, at the 78th United Nations General Assembly, a multi-stakeholder hearing on AMR was convened as part of the preparatory process of the Second High-Level Meeting on AMR. The hearing was supported by WHO, the Food and Agriculture Organization of the United Nations, the United Nations Environment Programme, the World Organisation for Animal Health (also known collectively as the Quadripartite organizations) and other relevant partners [31]. As a result of intergovernmental negotiations, in September 2024, Heads of State signed a political declaration. The need to scale up multisectoral, cross-sectoral and inter-disciplinary efforts and the engagement of all relevant sectors was recognized, where there is a call for generating an effective whole-of-government and whole-of-society response, including a One Health approach [32].

WHO established the One Health Initiative to unify efforts in addressing human, animal, and environmental health across the organization. This integrated approach acknowledges the interconnectedness and interdependence of the health of people, animals, and ecosystems, with the goal of sustainably enhancing global health security. By engaging various sectors, disciplines, and communities across different levels of society, the initiative aims to promote well-being and address the shared challenges to health and ecosystem sustainability.However, the progress in implementing integrated cross-domain solutions or strategies has not been evident, despite of its critical importance in accelerating progress, maximizing impact, and ensuring the sustainability of efforts to end the epidemics of HIV/AIDS, tuberculosis and AMR, especially in the context of limited funding. Integration of policies of Tuberculosis, HIV and antimicrobial resistance is similar to the problem of the integration management of co-morbidity or co-infection. A large number of comorbidity studies have been conducted by researchers in many fields, which provided an intentional reference for this study [33–49].

Overall, the integrated management of co-morbidity or co-infection has been evolving, with ongoing research aimed at improving patient outcomes through better understanding, treatment, and care coordination. To achieve the goal, a theoretical framework is required to address the complexity of multiple conditions in a single patient from a health system view. The Social Ecological Model will be a good theoretical framework that could be used to address the complexity of multiple conditions in a single patient. This model emphasizes that individual behavior and health are influenced by multiple dimensions, including individual, interpersonal, community, organizational, and social factors, and is widely used in the fields of health promotion, disease prevention, and public health with a primary focus on understanding the multilayered factors that affect an individual’s health and well-being [50, 51]. The challenges of TB, HIV, and microbial drug resistance in a single patient could also be better addressed based on this model. We selected TB, HIV, and AMR based on the following criteria: (i) cross-cutting policy salience and extensive World Health Organization (WHO) governing body output, enabling comparative analysis; (ii) documented comorbidity and care cascade intersections (e.g., TB/HIV coinfection) alongside shared delivery platforms (diagnostics, sample referral networks, antimicrobial stewardship); (iii) distinct yet complementary governance and financing architectures that challenge integration efforts (e.g., Global Fund TB/HIV grants; Quadripartite collaboration on One Health for AMR); and (iv) availability of comparable indicators for national adoption and impact monitoring. While alternative triads exist, TB, HIV, and AMR encompass complementary yet intersecting transmission ecologies, rendering them an incisive test case for WHOs policy integration capacity. TB transmission is primarily airborne (via droplets/aerosols), readily amplified in congregate and healthcare settings, necessitating triage protocols, ventilation systems, respiratory protection, and rapid diagnostics. HIV transmission occurs through sexual exposure, blood/injection exposure, and vertically (mother-to-child), requiring comprehensive prevention packages (condoms, pre-exposure prophylaxis/post-exposure prophylaxis [PrEP/PEP], harm reduction, blood safety) and chronic care management—often within facilities concurrently managing TB. AMR, distinct from a single pathogen, involves the dissemination of resistant organisms and genes across interconnected pathways, including healthcare settings, communities, food-animal production, and environmental reservoirs (water/wastewater), driven primarily by antimicrobial use and healthcare-associated infections; its mitigation necessitates antimicrobial stewardship, infection prevention and control (IPC), and multisectoral One Health approaches. These transmission routes converge operationally: individuals living with HIV exhibit heightened susceptibility to TB;TB and HIV drug resistance is a major contributor to AMR;inadequate IPC escalates risks of both nosocomial TB transmission and AMR spread; shared laboratory networks, sample referral systems, and supply chains underpin responses to all three challenges. As the requisite control measures span multiple socio-ecological levels, concurrent assessment of TB, HIV, and AMR reveals critical junctures where WHO guidance can—and should—be integrated across distinct transmission pathways.

The WHO benchmark actions have filled a key gap in implementation guidance to address TB, HIV, and microbial drug resistance problems in many countries [52]. The WHO is at the forefront of global health policy development, and its documents offer a comprehensive and consistent data-set for analyzing the integration of TB, HIV, and AMR within the global health framework. However, The World Health Organization (WHO) still should coordinate multiple departments and multi-organization collaboration to adopt an integrated strategy based on the Social Ecological Model to simultaneously address TB, HIV, and microbial drug resistance problems, thereby improving the efficiency and effectiveness of relevant programs under limited resources and funding after COVID-19. An integrated strategy in services, individual, interpersonal, community, social, and policy factors will help solve the problem of nonconformity and incomplete integration in TB, HIV, and AMR. This study will assess to what extent WHO resolutions and decisions on TB, HIV, and microbial drug resistance have worked from the social-ecological perspective.

While the action plans and strategies of organizations other than WHO are undoubtedly valuable and complementary. For example the Joint United Nations Programme on HIV/AIDS (UNAIDS), the Global Fund to Fight AIDS, Tuberculosis, and Malaria, Stop TB Partnership, Food and Agriculture Organization (FAO), World Organisation for Animal Health (WOAH, formerly OIE), United Nations Environment Programme (UNEP) and many others. Future research could consider a comparative analysis involving multiple international organizations to provide a broader perspective on global health strategies. For our study we take the example of three international organizations each at the forefront of TB, HIV and AMR, namely the Stop TB Partnership (Stop TB), the Joint United Nations Programme on HIV and AIDS (UNAIDS), and the United Nations Environment Programme (UNEP) to analyze their development and implementation of action plans for TB, HIV, and AMR from the social-ecological perspective.These organizations were chosen due to their prominent leadership and influence in their respective fields, their comprehensive and multidisciplinary approaches to policy-making and implementation, and their extensive collaboration across sectors. Stop TB Partnership leads global efforts in TB control, UNAIDS plays a crucial role in HIV prevention and treatment, and UNEP focuses on the environmental dimensions of AMR. Each organization provides action plans and policy documents, making them ideal case studies for our research.

This study examines the integration of tuberculosis (TB), human immunodeficiency virus (HIV), and antimicrobial resistance (AMR) within World Health Assembly (WHA) resolutions and Executive Board (EB) decisions. Utilizing the socio-ecological model (SEM) [50, 51], which conceptualizes multi-level health determinants—encompassing individual, interpersonal, community, organizational, and societal factors—this analysis elucidates the World Health Organization’s (WHO) strategic orientation and prospective impact. Herein, ‘integration’ denotes service delivery arrangements combining interventions, platforms, or functions across distinct disease domains. SEM is applied to health determinants and actors, while ‘integration’ pertains specifically to service design, implementation, governance and accountability mechanisms. Vertical programs represent disease-specific delivery mechanisms, financing streams, and related structures. Our analytical framework engages the vertical–horizontal continuum and the diagonal approach, which strategically utilizes disease-targeted investments to strengthen health systems [53–56]. While TB, HIV, and AMR have historically been addressed through largely vertical programs, the socio-ecological lens offers a structure to surface cross-cutting enablers that can support integrated delivery. Building on this perspective, our study not only maps disease-specific policy architectures but also tests whether common themes emerge across levels (individual/community, service/health-system, policy, and global). We hypothesized that such themes would signal concrete entry points for integration and will yield recommendations for fortifying cross-cutting strategies, augmenting multisectoral cooperation, and expediting responses to global health threats.

## Methods

### Study design

We performed a mixed-methods policy analysis, combining quantitative content analysis of policy documents with qualitative thematic analysis, framed by a social–ecological model. The social–ecological model allowed us to examine policy integration at multiple levels (individual, interpersonal, community, institutional, and societal).

### Data collection

We introduced a structured taxonomy of World Health Organization (WHO) governing body documents and related outputs. In this study, documents are categorized as: (1) World Health Assembly (WHA) Resolutions (normative, agenda-setting instruments requesting or urging action by Member States and the WHO Secretariat), (2) Executive Board (EB) Decisions and EB Reports/Annexes (agenda-preparatory and oversight instruments operationalizing strategic directions into follow-up requests), (3) Secretariat/Technical Reports to the WHA/EB (analytical or programmatic updates guiding subsequent decisions), and (4) WHO Technical/Normative Guidance (e.g., consolidated guidelines, policy briefs, implementation manuals). For each category, the following dimensions were mapped: (a) policy function; (b) typical implementation pathway (global → regional → national program planning and financing → service delivery); (c) primary target audiences and user communities (ministries of health and finance, national TB/HIV/antimicrobial resistance (AMR) programs, regulators, procurement bodies, clinical networks, civil society); (d) funding implications (domestic budgets, alignment with the Global Fund/President’s Emergency Plan for AIDS Relief (PEPFAR), AMR Multi-Partner Trust Fund (MPTF)/One Health windows); and (e) outcome/impact indicators utilized by Member States and partners. Details are provided in Table 1.

**Table 1:** Taxonomy of WHO governing-body documents and linked functions, pathways, audiences, funding, and indicators

Category	Policy function	Implementation pathway	Target audiences/users	Funding implications	Outcome/impact indicators
WHA Resolution	Sets global priorities; requests actions; establishes accountability expectations.	Adopted by the World Health Assembly → subsequently informs regional committee guidance → subsequently integrated into national strategies, annual operational plans, and partner grants.	Ministry of Health and Ministry of Finance leadership; national-level initiatives; legislative bodies; collaborating organizations.	Communicates strategic priorities to donor entities; facilitates domestic fiscal budgeting processes; and contributes to advancing Universal Health Coverage (UHC) reforms through strategic alignment with Global Fund grant-making mechanisms.	Inclusion within national strategic frameworks; establishment of dedicated budget al.locations; integration of coverage and quality indicators into Health Management Information Systems (HMIS); and periodic submission of progress reports to the World Health Assembly (WHA).
EB Decision/Report	Prepares WHA agenda; requests Secretariat follow‑up; monitors progress.	Executive Board (EB) follow-up activities → Secretariat technical products → country-level technical assistance delivered through regional/country offices.	WHO regional/country offices; national focal points; technical partners.	Oversees the Secretariat’s Technical Assistance resources and formulates joint work plans with partners.	All requested deliverables have been satisfactorily completed; technical assistance has been duly provided; project milestones specified by the Executive Board have been successfully attained.
Secretariat/Technical Report	Synthesizes evidence; proposes options; provides status updates.	Informs EB/WHA deliberations while also being cited by national-level guideline committees.	National technical working groups; clinicians; regulators.	Supports donor concept notes; informs procurement forecasts.	Citations in national guidance; procurement changes; monitoring dashboards updated.
WHO Technical/Normative Guidance	Defines standards and good practices; operationalizes policies.	Adopt or adapt measures at the national level through Technical Working Groups (TWGs), and disseminate these to facility-level standard operating procedures (SOPs) and training programs.	Clinical networks; laboratory systems; supply chains; civil society organizations (CSOs).	Determines commodity/training budgets; supports pooled procurement.	Facility-level implementation; adherence and audit outcomes; clinical endpoints (e.g., antiretroviral therapy (ART) initiation rates among tuberculosis patients; antibiotic consumption quantified in defined daily doses [DDD]).

Due to the key role played by World Health Assembly (WHA) resolutions and Executive Board (EB) decisions/reports in guiding global integration practices, we systematically reviewed these policy documents issued between January 2015 and February 2024, sourced exclusively from WHO databases. Quantitative trend analysis was further conducted using data on national and regional policy documents pertaining to tuberculosis (TB), human immunodeficiency virus (HIV), and antimicrobial resistance (AMR), extracted from the Cortellis Regulatory Intelligence database (Clarivate) [57]. Cortellis Regulatory Intelligence is a subscription-based global database that aggregates and curates official requirements, guidance documents, regulatory decisions, and historical precedents issued by national and regional authorities for medicines, biologics, medical devices, and in vitro diagnostics (IVDs). The platform provides English-language summaries, cross-market comparative analyses, real-time regulatory alerts, and expert insights to support regulatory strategy development, submission processes, post-approval change management, and compliance monitoring. As of 2025, it monitors evolving regulatory frameworks across 75 jurisdictions(details presented in appendix), offering over 2,000 structured summaries and more than 43 comparative views derived from a corpus of approximately 285,000 regulatory documents and over 7,000 expert reports [57]. Country details are presented in the appendix. The inclusion criteria encompassed documents addressing TB, HIV, or AMR (or any combination thereof) at the global policy level. Documents unavailable in English or lacking direct relevance to these specified health issues were excluded.

During this period, we established a standardized set of search terms for each disease, encompassing variations of disease names, abbreviations, and associated terminology. For tuberculosis, the search terms employed were: “tuberculosis”, “TB”, “Mycobacterium tuberculosis”, and “multidrug-resistant tuberculosis”. For HIV/AIDS, the terms utilized were: “HIV”, “AIDS”, “human immunodeficiency virus”, and “acquired immunodeficiency syndrome”. For antimicrobial resistance, the search terms included: “microbial resistance”, “antimicrobial resistance”, and “AMR”.Furthermore, the Joint United Nations Programme on HIV and AIDS (UNAIDS), the Stop TB Partnership (Stop TB), and the United Nations Environment Programme (UNEP) are pivotal in shaping global policy decisions concerning HIV, tuberculosis, and antimicrobial resistance. These organizations frequently collaborate with the World Health Organization (WHO) to formulate prevention and control policies addressing these public health challenges; details are presented in Table 2.This study also incorporated the action plans and policy recommendations outlined in the most recent reports from UNAIDS, Stop TB, and UNEP on HIV, TB, and antimicrobial resistance [58–60]. This analysis complements the comparison of WHO-organized action plans and strategies, facilitating the identification of trends and gaps in policy integration for these critical health concerns.

**Table 2:** Coordination architecture across WHO, stop TB, UNAIDS, and UNEP

Institution	Core mandate	Collaboration instruments	Resource sharing	Joint M&E/conflict resolution
WHO	Normative leadership; guidance; coordination	World Health Assembly/Executive Board; joint work plans; regional committees; Country Coordination Mechanism participation	Technical Assistance budgeting; pooled procurement platforms; joint review mechanisms	Standardized performance indicators; collaborative progress reports; and escalation mechanisms via EB/WHA reporting.
Stop TB	Global TB partnership and advocacy/brokering	Partnership Board and Its Working Groups: Alignment with the Global Fund	Catalytic funding; market-shaping	Scorecards, Grant KPI Alignment, Partner Forums
UNAIDS	HIV strategy, convening, and accountability	Printed Circuit Board (PCB); Joint Programme; Country Convening	Co-funded budgets; collaborative monitoring and evaluation (M&E) systems.	Global AIDS Surveillance: National Review Missions
UNEP	Environmental determinants and AMR within One Health	Quadripartite One Health Joint Plan of Action	One Health trust funds; joint projects	Shared One Health Indicators: Interagency Task Forces

Our study employs a socio-ecological framework to examine policy integration, evaluating the operationalization of WHO guidance across individual, facility/organizational, community, and policy–environment levels. Unlike tuberculosis (TB) and HIV—which are primarily grounded in clinical and public health service delivery—antimicrobial resistance (AMR) inherently involves environmental transmission pathways (e.g., pharmaceutical and hospital effluents, wastewater, agricultural use and runoff, contaminated surfaces and water systems). The United Nations Environment Programme (UNEP) constitutes the environmental pillar of AMR governance, providing essential regulatory, surveillance, and stewardship mechanisms beyond the purview of health ministries, necessary for curbing the dissemination of resistant organisms and genes. Consequently, the inclusion of UNEP (i) aligns with our framework’s cross-sectoral scope; (ii) enables the analysis of inter-organizational coordination, specifically the interaction of health guidance with environmental standards, waste-management policies, and pollution controls; and (iii) reveals operational interfaces with TB/HIV programs—notably infection prevention and control (IPC), water, sanitation and hygiene (WASH) in health facilities, laboratory biosafety, and shared sample-referral and waste streams—where inadequate environmental controls can concurrently drive nosocomial TB transmission and healthcare-associated AMR.However, the WHO Global Action Plan on Antimicrobial Resistance may provide a more focused framework for examining the health-specific integration of AMR, though UNEPs environmental governance remains a critical element of AMR control.

### Content analysis

We first analyzed WHO’s published resolutions and EB decisions/annexes from 2015 to 2024. In total, 130 WHO governing body documents (WHA resolutions and EB decisions) were identified in this period. To analyze TB, HIV and AMR content within these documents, we developed standardized search terms for each topic and performed a word frequency analysis to quantify mentions of “tuberculosis”, “HIV”, “antimicrobial resistance”, and “One Health.” We also noted occurrences where all three terms appeared together, indicating integrated discussion.For the broader policy landscape, we collected metadata from Cortellis on policy/regulatory documents (e.g. guidelines, strategy plans, regulatory announcements) related to TB, HIV, and AMR globally from 2015 to 2024. We tallied the number of documents per year, by document type, and by issuing country/region, to assess trends in policy activity and the breadth of country coverage for each disease area.

### Thematic analysis

To analyze the descriptions related to TB, HIV, and antimicrobial resistance from WHA resolution and EB decisions, we developed a thematic analysis framework containing sections of specific document titles, integration context, proposed action, governance, and accountability mechanisms. In addition to disease-specific coding, we inductively tagged cross-cutting enablers that appeared across TB, HIV, and AMR at each socio-ecological level,which is analyzed in multiple dimensions, including individual, interpersonal, community, organizational/institutional, and societal factors. We then synthesized these themes in a comparative matrix to identify practical entry points for integrated policy and program design.A group of researchers independently reviewed the documents and extracted the relevant descriptions and categorized them into different themes. The extracted descriptions were compiled into various tables and were analyzed using quantitative and qualitative methods to identify patterns and gaps in integration.We also reviewed literature and case studies (identified via PubMed and organizational reports) that demonstrate integrated TB/HIV or TB/HIV/AMR interventions to inform the results,discussion and recommendations.

### Analysis

Quantitative analysis describes the prevalence of integrated descriptions,policy trends among different countries between the three topics, the frequency of keywords TB, HIV, and AMR were calculated through the keyword search function of Microsoft Word and the Summary function of Excel,a venn analysis of jurisdictional coverage in TB, HIV, and AMR policy documents. Qualitative analysis identifies thematic coding of challenges, solution strategies, and integrated content based on the social ecological model through NVivo11. Furthermore, the Joint United Nations Programme on HIV and AIDS (UNAIDS), the Stop TB Partnership (Stop TB), and the United Nations Environment Programme (UNEP) play a significant role in shaping global policy decisions related to HIV, tuberculosis, and microbial antimicrobial resistance. These organizations often collaborate with the World Health Organization (WHO) to develop prevention and control policies for these issues. In this study, we conduct a thematic framework analysis of the action plans and policy recommendations outlined in the most recent reports from UNAIDS, Stop TB, and UNEP on HIV, TB, and antimicrobial resistance [58–60]. This analysis complements the comparison of action plans and strategies organized by WHO, helping to identify trends and gaps in policy integration for these important health concerns.This approach provides a comprehensive and nuanced understanding, generates reliable and meaningful research findings, and provides recommendations for strengthening integration in global health strategies. By reviewing the patterns, integration, and gaps in the WHO policy document, we provide evidence-based insights to guide future policy development and implementation.We also triangulated findings from the WHO document analysis with external evidence from case studies and program evaluations to ground our recommendations in real-world experience.

## Results

### Quantitative results

#### Overview of the inclusion documents

Our comprehensive search and screening in WHO databases captured 28 relevant documents, including 11 WHA resolutions,17 EB decisions and annexes covering global health issues, particularly tuberculosis, HIV and antimicrobial resistance. The specific issues related to tuberculosis, HIV and antimicrobial resistance among the 28 documents are summarized in Table 3. TB has 3 resolutions and 5 EB decisions; HIV has 1 resolution and 3 EB decisions, which is the least issues; AMR has 4 resolutions and 7 EB decisions, which owns the most attention compared with TB and HIV.

**Table 3:** WHO resolutions and decisions for TB, HIV and AMR since 2015

Year	TB	HIV	AMR
2015			A68/20、EB136/19、EB136/20
2016		A69/31、EB138/29	A69/24、EB138/24
2017			A70/12、EB140/11
2018	A71/16、A71/15、EB142/16		
2019	A72/20、EB144/21		A72/18、EB144/19
2020	EB146/10、EB146/11		
2021		EB148/37	EB148/11
2022	EB150/9	EB150/8	
2023			
2024 (February)	EB154/10		EB154/13

#### The frequency of the keywords

The keywords frequencies of tuberculosis, HIV, antimicrobial resistance and one health in different resolutions and decisions are summarized in Table 4. The keyword “Tuberculosis” appears with varying frequencies across documents. For instance, it is mentioned 167 times in document EB150/9, which is the highest count for TB in the provided data. However, in documents EB146/11 and EB148/11, TB is not mentioned at all. The keyword “HIV” shows a high count in document A69/31 with 590 mentions, indicating a strong focus on this topic in that particular resolution or decision. Document EB138/29 also has a significant number of mentions (111). Conversely, there are documents like EB148/37 and EB150/8 with relatively fewer mentions. The keyword “antimicrobial resistance” is most frequently mentioned in document EB136/20 with 111 occurrences, suggesting a concentrated discussion on the topic in that document. Document A68/20 also has a high count of 115 mentions. However, in documents like EB148/11 and EB154/13, the keyword is mentioned only once. The co-occurrence is relatively low, with the highest being in document A68/20, EB136/20，and EB140/11, where it happens twice, and several documents show no instances of all three keywords appearing together. The data suggests that the focus on each keyword varies significantly across different resolutions and decisions, which reflects the priorities and topics of discussion during the respective sessions. The high frequency of mentions for a particular keyword in some documents could indicate a stronger emphasis on that health issue during those sessions. The lack of co-occurrence of all three keywords and one health in many documents might suggest that while these issues are discussed separately, there may be opportunities for more integrated discussions that consider the interrelated nature of these global health challenges.

**Table 4:** The frequency of the keywords of TB, HIV and AMR appeared in different global health issues

Documents		TB	HIV	AMR	One Health	Three key words appeared in one paragraph
TB	A71/16	34	2	3	0	1
	A71/15	8	6	1	0	1
	EB142/16	34	2	3	0	1
	A72/20	78	4	3	0	1
	EB144/21	73	4	3	0	1
	EB146/10	89	10	2	1	0
	EB146/11	67	0	3	0	0
	EB150/9	167	9	0	0	0
	EB154/10	105	8	1	0	0
HIV	A69/31	34	590	1	0	0
	EB138/29	5	111	0	0	0
	EB148/37	4	35	0	0	0
	EB150/8	5	42	1	0	0
AMR	A68/20	7	4	115	0	2
	EB136/19	1	1	26	0	1
	EB136/20	7	4	111	0	2
	A69/24	2	2	20	2	1
	EB138/24	0	0	12	0	0
	A70/12	10	5	31	2	1
	EB140/11	6	4	17	1	2
	A72/18	12	15	70	4	1
	EB144/19	12	12	47	3	1
	EB148/11	0	0	53	7	0
	EB154/13	1	1	109	2	1

#### Broader policy trends (2015–2024)

**Fig. 1:**
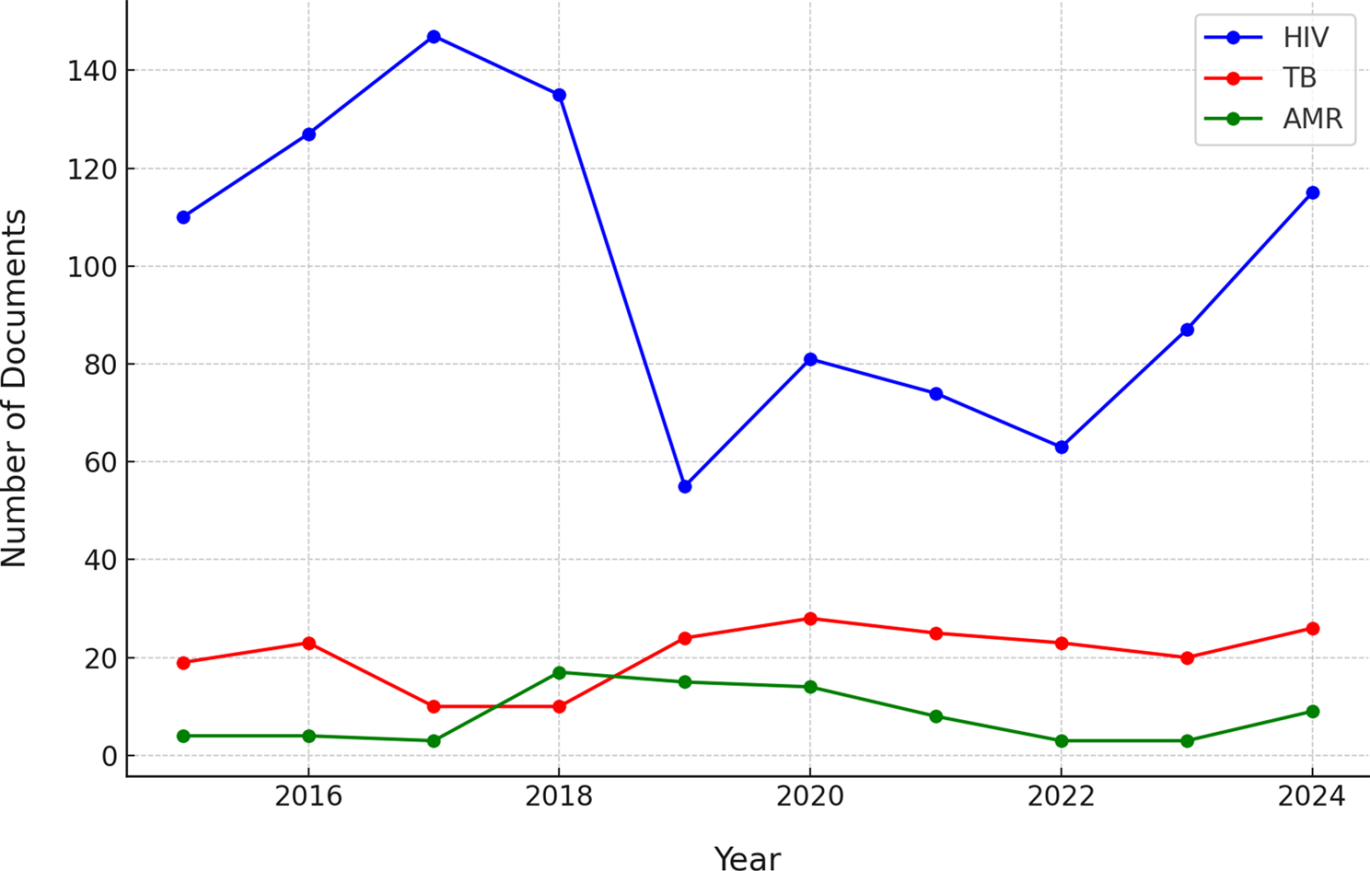
Annual number of policy/regulatory documents related to TB, HIV, and AMR worldwide from 2015 through 2024

In addition to WHO resolutions and decisions, we examined the broader landscape of policy documents pertaining to tuberculosis (TB), HIV, and antimicrobial resistance (AMR) issued globally between 2015 and 2024. Analysis of the Cortellis regulatory intelligence database revealed divergent trends in the volume of policy activity for each issue (Fig. 1). HIV-related policy and regulatory documents were issued in substantially greater numbers annually compared to those addressing TB or AMR. HIV documents peaked in 2018 with 150 issued that year, driven by numerous approvals of antiretroviral drugs, guidelines, and program policies; subsequently, issuance declined slightly and stabilized at 90–110 documents annually through 2024. TB-related policy documents were considerably fewer per year (typically 7 to 26), but exhibited surges in 2019 and again during 2023–2024, potentially corresponding to renewed global TB commitments and the introduction of new diagnostic or treatment guidelines. AMR-related policy documents were minimal prior to 2017, then increased, with a notable peak in 2022 when approximately 30 AMR policy documents were issued globally. The cumulative volume over the decade underscores a stark imbalance: 1,103 HIV-related policy documents versus 144 for TB and 120 for AMR. This pattern illustrates that HIV has been the subject of far greater policy and regulatory activity, reflecting its longstanding prioritization on the global health agenda.The synchronized 2019 peaks for HIV and TB (and sustained high HIV output in 2024–25) may reflect broader policy windows (e.g., waves of approvals/generics/indications or ecosystem changes). These windows can accelerate cross-national alignment through reference or reliance pathways.

**Fig. 2:**
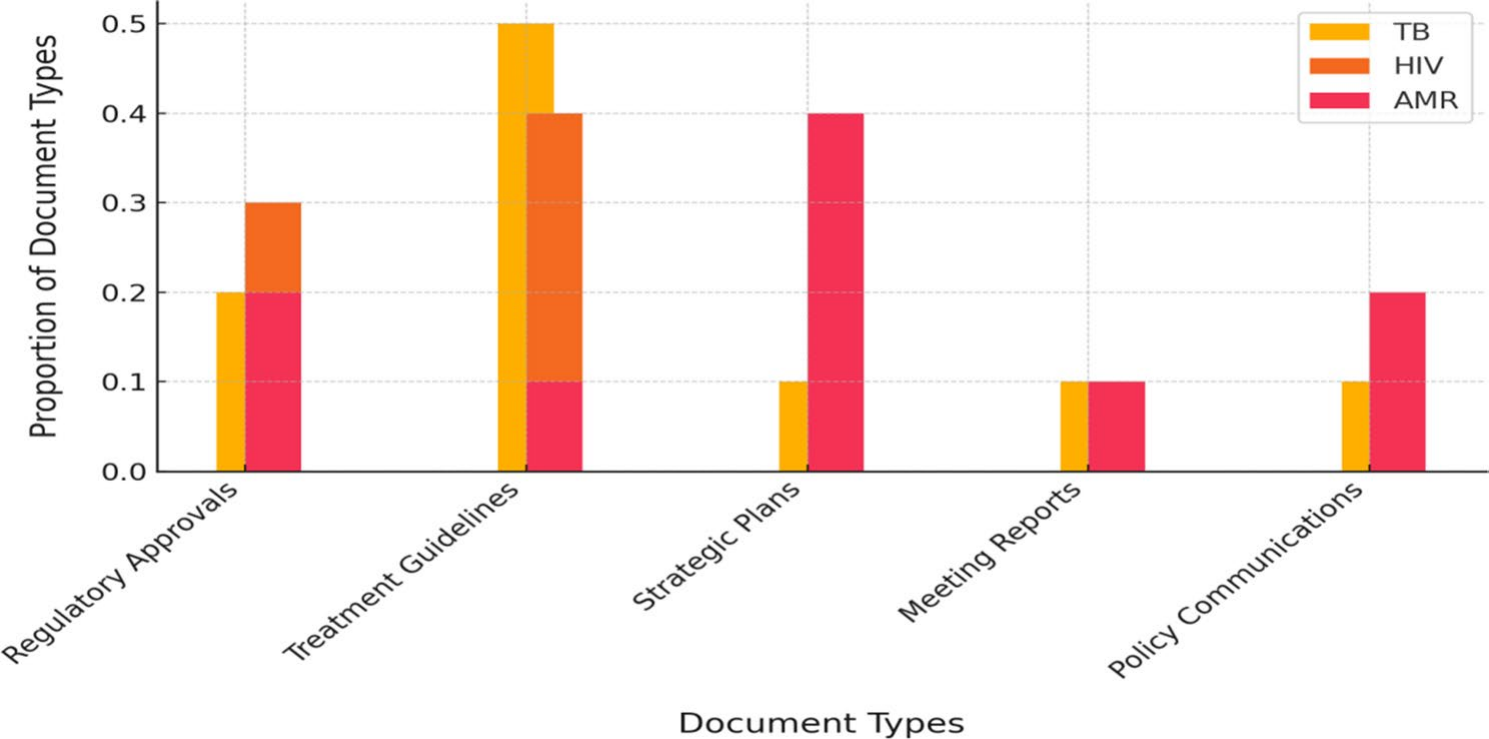
Distribution of document types for TB, HIV, and AMR policy documents (2015–2024)

A comparative analysis of document categories revealed significant differences in **the nature of policy documents across disease areas** (Fig. 2). For HIV and tuberculosis (TB), a substantial proportion of documents comprised **regulatory approvals and treatment guidance.** Examples include U.S. Food and Drug Administration (FDA) supplemental new drug approvals, European Medicines Agency (EMA) opinions, and clinical guidelines, reflecting ongoing updates to therapeutic and diagnostic interventions for these diseases. Specifically, 35% of HIV-related documents were FDA supplemental new drug approvals (frequently concerning updates to antiretroviral indications), while 11% constituted press releases typically associated with such approvals. TB documents further encompassed regulatory actions, such as new TB drug approvals or orphan drug designations, which accounted for 17% of TB documents, alongside national TB program guidelines. In contrast, antimicrobial resistance (AMR)-related policy documents were predominantly **strategic and collaborative in** nature. The largest single category (28%) comprised records of **multi-stakeholder meetings** (e.g., One Health AMR coordination meetings and network reports), followed by policy reports, official communications, and governmental announcements of AMR action plans. Documents pertaining to product approvals were notably scarce within the AMR category. These patterns indicate that AMR policy efforts have primarily centered on strategic development and coordination, frequently within One Health frameworks, rather than regulating new therapeutics or establishing clinical guidelines.

The **geographical** scope of policy documents varied across the three domains. HIV-related policies issued between 2015 and 2024 encompassed **38 distinct jurisdictions**, indicating extensive global engagement (including numerous high- and middle-income countries issuing HIV guidelines or regulatory approvals, alongside global/regional bodies). Tuberculosis (TB)-related documentation covered **29 countries/regions**, demonstrating moderately reduced diversity yet maintaining a multi-continental distribution (predominantly initiated by the United States and European Union, with additional contributions from countries such as Thailand, China, South Africa, and Brazil). Antimicrobial resistance (AMR)-related policy instruments originated from merely **13 jurisdictions** during the decade—primarily the European Union, United States, Canada, India, and a limited number of additional jurisdictions—reflecting the concentration of formal AMR policy initiatives (within the Cortellis-defined dataset) within specific regions. The EU and USA dominate across domains, acting as agenda-setting hubs.

**Fig. 3:**
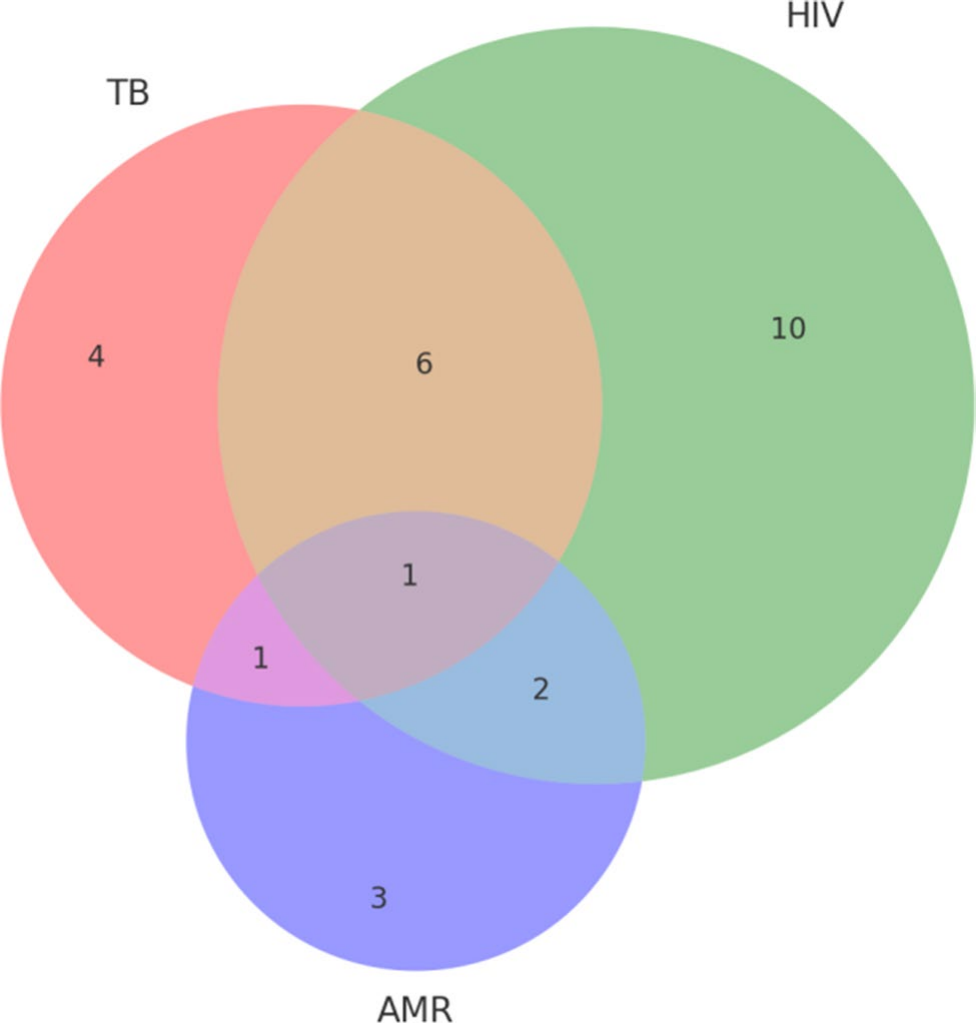
Overlap of jurisdictions involved in TB, HIV, and AMR policy documents (2015–2024)

A Venn analysis of jurisdictional coverage (Fig. 3) reveals that while a core group of jurisdictions (United States, European Union, India, China, South Korea, Kenya) have established policies addressing all three domains, policy efforts in numerous countries remain siloed. Notably, several countries (e.g., Russia, Greece, Philippines, Bulgaria) appeared exclusively within TB policy datasets, while others (e.g., Singapore, Chile, Switzerland, Australia) were documented solely within HIV policy datasets. Conversely, **AMR policy activity was almost entirely confined to high-income settings** (with jurisdictions such as Ireland, Sweden, and Germany appearing uniquely within AMR datasets). This distribution indicates that integrated policy attention to TB, HIV, and AMR collectively is predominantly confined to a limited number of jurisdictions, with most countries continuing to focus policy development on singular issues rather than adopting a combined approach.

The above quantitative findings underline that **policy integration is not yet evident in the volume or breadth of global policy outputs** – HIV has commanded the most policy attention, and AMR the least, with limited convergence. These data reinforce the need for greater integrated policy frameworks that can be adopted across more countries.

### Qualitative results

#### Comparative analysis of coping strategies for TB, HIV, and AMR

The thematic framework analysis of the WHOs recent resolutions and decisions on tuberculosis, HIV, and antimicrobial resistance was detailed in Table 5 based on the social ecological model. The TB action plan focuses on improving coverage of treatment, rapid diagnostic tests, and increasing investment in TB research. This includes realigning strategies and targets at the country level, global collaboration, community engagement, and scaling up health services, especially for children and people with drug-resistant TB. The AIDS action plan focuses on strengthening national ownership of the health agenda, the role of community organizations, and WHOs role in strategic leadership, partnerships, and public health advocacy. It also highlights the use of primary health care platforms and the strategic integration of disease-specific and shared approaches based on country-specific contexts and health system capacities. The action plan on microbial resistance focuses on preventing all infections that can lead to antibiotic use, ensuring universal access to quality diagnosis and appropriate treatment, and enhancing strategic information and innovation. This involves the effective implementation of national action plans and the management of antibiotic use outside the health system. In summary, the action plans in all three areas emphasize leadership and ownership at the national level, global and regional cooperation, community engagement and the role of civil society organizations, and the importance of research and innovation. The difference is that the strategy for each disease area is tailored to its specific public health needs and challenges with targeted goals and approaches. Strategies for TB and HIV focus more on disease-specific treatment and prevention measures, while strategies for microbial resistance focus on cross-sectoral collaboration and the rational use of antimicrobials. In addition, monitoring and reporting mechanisms are used to ensure the effective implementation and continuous improvement of these action plans.

**Table 5:** WHO action plans for TB, HIV and AMR

Areas of action plans	TB	HIV	AMR
Individual level	• Improve the diagnosis and treatment of TB • Promote TB prevention measures, such as vaccination	• Emphasis on the prevention, diagnosis, and treatment of HIV • Promotion of ART and preventive measures	• Raise awareness of the rational use of antimicrobial agents • Promoting the role of individuals in preventing drug resistant infections
Interpersonal level	• Community involvement and TB education • Reducing discrimination and stigma against TB patients	• Community based HIV support and education • Role of community involvement in HIV prevention and treatment	• Community education and participation to reduce the inappropriate use of antimicrobial agents
Community level	• Community based TB prevention and control programs • Community mobilization to improve coverage of TB services	• Community based HIV prevention and treatment programs • Community engagement to improve accessibility to HIV services	• Community based antimicrobial resistance education and prevention programs
Institutional level	• TB diagnosis and treatment services provided in medical institutions • Strengthen infection control measures in medical institutions	• Medical institutions provide comprehensive HIV services • The role of healthcare institutions in HIV prevention and treatment	• Role of healthcare institutions in the management of antimicrobial resistance • Antimicrobial use policy in healthcare settings
Societal level	• National TB control policy • International cooperation and financial support • Provide integrated TB prevention, diagnosis, treatment and rehabilitation services -Collaboration across sectors to integrate TB services	• National HI V/A IDS policy • Global health sector strategy • Provide integrated HIV prevention, treatment, care and support services • Cooperate across departments to integrate HIV/A IDS services	• National Action Plan against antimicrobial Resistance • International cooperation and regulatory framework • Provide antimicrobial resistance prevention, diagnosis, management and education services • Cross-departmental collaboration to integrate antimicrobial resistance services

A thematic framework analysis based on the social-ecological model of the recent WHO governance and accountability mechanisms for TB, HIV, and antimicrobial resistance was detailed in Table 6. TB, AIDS, and microbial antimicrobial resistance demonstrate common governance principles and accountability mechanisms in governance and accountability strategies, including strategic leadership, partnership building, public health advocacy, norms and standards setting, technical support and capacity building, global monitoring and reporting, and the importance of funding. Nonetheless, strategies for each disease domain also set different disease-specific goals and implementation priorities based on their specific disease characteristics and global health challenges. For example, TB and AIDS may focus more on disease-specific treatment and prevention, while microbial resistance emphasizes cross-sectoral collaboration and rational use of anti-microbial agents. Furthermore, the focus of research and innovation, ways of community engagement, and specific implementation details of the accountability framework were also tailored to the global strategies and goals of each disease. The similarities and differences between these strategies reflect the flexibility and adaptability of global health in responding to different diseases while emphasizing the importance of cross-domain cooperation and common goals to ensure an effective response to global health challenges.

**Table 6:** Governance and accountability mechanisms for the WHO response to TB, HIV, and AMR

Areas of action plans	TB	HIV	AMR
Individual level	• Participation and feedback mechanisms of patients and affected populations • Individual adherence to TB treatment and self-management	• Participation of the patients and the affected populations • Individual compliance to HI V/A treatment of IDS and self-management	• Personal awareness and behavior change of rational use of antimicrobial agents
Interpersonal level	• Family and community support networks • To reduce discrimination and stigma against TB patients	• Family and community support networks • Reduce discrimination and stigma of people with HIV/A IDS	• Community education and participation to increase awareness of anti-microbial resistance
Community level	• Community involvement and leadership of the TB control program • Community supervision and evaluation service provision	• Community involvement and leadership of the HI V/A IDS prevention program • Community supervision and evaluation service provision	• Community involvement and leadership of antimicrobial resistance programs • Community supervision and evaluation of antimicrobial agent use
Institutional level	• Governance structure of healthcare institutions and public health systems • Ensure the quality of TB services	• Governance structure of healthcare institutions and public health systems • Ensure the quality of HI V/A IDS services	• Governance structure of healthcare institutions and public health systems • Ensure rationality of use of antimicrobial agents
Societal level	• National TB policy and strategy • International cooperation and financial support • Provide integrated TB prevention, diagnosis, treatment and rehabilitation services • Cross-sectoral collaborative integration services • Leadership and coordination of WHO and other partners • Periodically report and assess progress in TB control • Accountability and transparency at the national and international levels • Use of monitoring and evaluation data to guide policies and practices	• National HI V/A IDS policy and strategy • Global Health sector strategy • Provide integrated HI V/A IDS prevention, treatment, care and support services • Collaborative integrated services across departments • Leadership and coordination of WHO and other partners • Periodically report and evaluate the HI V/A IDS response progress • Accountability and transparency at the national and international levels -Use of monitoring and evaluation data to guide policies and practices	• National AMR policy and strategy -International cooperation and regulatory framework • Provide antimicrobial resistance prevention, diagnosis, management, and education services • Cross-collaborative integration services • Leadership and coordination of the WHO and other partners • Regular reporting and assessment of antimicrobial resistance response progress • Accountability and transparency at the national and international levels • Use of monitoring and evaluation data to guide policies and practices

#### Action plans of stop TB, UNAIDS, and UNEP

Stop TB, UNAIDS, and UNEP, along with WHO, are the most influential international institutions in the field of tuberculosis, AIDS, and antimicrobial resistance (AMR) challenges. Their action plans and governance accountability mechanisms have the potential to impact the development of future resolutions by WHO. A comparative analysis of Stop TB, UNAIDS, and UNEP in their TB, AIDS, and AMR action plans is presented in Table 7. The action plans for tuberculosis, HIV, and antimicrobial resistance all highlight the significance of political leadership, legal and policy reforms, sufficient funding, community involvement, prevention and education efforts, treatment and care promotion, monitoring and evaluation systems, research and innovation, and international cooperation. These programs form a comprehensive public health response framework aimed at enhancing disease management, promoting health equity, reducing disease burden, and achieving global prevention goals.

**Table 7:** Action plans for stop TB, UNAIDS and UNEP to address TB, HIV and AMR respectively

Areas of action plans	TB	HIV	AMR
Individual level	• Public health promotion, school education, and community seminars	• Education and publicity to improve health literacy	• Medical professional education, patient education
Interpersonal level	• Community mobilization activities, support groups, and peer education	• Partner education, psychological counseling	• Medical team training, patient communication training
Community level	• Community health centers, mobile clinics, and community volunteers	• Community organization and activities, volunteer mobilization	• Community education programs, and public awareness promotion activities
Institutional level	• Upgrading of health facilities, personnel training, and service process optimization	• Train the health workers and improve the facilities	• Medical institutions were policy formulated and infection control measures were strengthened
Societal level	• Policy advocacy, a regulatory framework, policy evaluation, and revision • International cooperation projects, financial assistance and technical exchanges	• Policy advocacy and multi-party cooperation • Strengthen international cooperation to improve the reporting, monitoring and regulatory systems • Environmental improvement, improved resource accessibility	• Policy and regulation formulation, and supervision of environmental emission standards • International cooperation projects, financial assistance and technical exchanges • Regulation of antibiotic use in agriculture and food production to reduce environmental antibiotic contamination

**Table 8:** Governance and accountability mechanisms for the Stop TB, UNAIDS and UNEP response to TB, HIV and AMR respectively

Governance and accountability mechanisms	TB	HIV	AMR
Individual level	• Health education and awareness promotion	• Individual behavior and responsibilities	• Knowledge of AMR by medical professionals and patients
	• Objective: To improve individual TB awareness and awareness of prevention	• Objective: Improve personal health awareness	• Objective: To improve the knowledge level of medical professionals and patients about AMR
	• Influencing factors: Cultural belief, education level and health literacy	• Impact actors: Lack of knowledge, behavior is difficult to change	• Impact actors: Insufficient awareness of AMR by medical professionals and patients
	• Implementation strategy: Public health promotion, school education, community seminars	• Implementation strategy: Education and behavior change programs	• Implementation strategy: Ensure that medical professionals follow the antibiotic use guidelines
	• Accountability mechanisms: Monitoring knowledge levels and behavior change rates	• Accountability mechanism: Monitoring the assessment of individual behavior change	• Accountability mechanism: Assess the knowledge levels of medical professionals and patients
Interpersonal level	• Community engagement and support	• Social networks and partnerships	• Communication within the medical team
	• Objective: To encourage community members to participate in TB control activities	• Objectives: HIV education and support among partners	• Objective: Enhance communication and collaboration within the healthcare team
	• Factors: Community leaders, social networks, and group dynamics	• Factors: Social barriers, lack of trust	• Factors: Insufficient communication within the medical team
	• Implementation strategies: Community mobilization activities, support groups, and peer education	• Implementation strategy: Social skills training, support groups	• Implementation strategy: Regularly assess teamwork and communication effectiveness
	• Accountability mechanisms: Monitoring of community engagement and support	• Accountability: Monitoring of social network strength and partner support	• Accountability mechanism: Monitoring the communication effectiveness of the medical team
Community level	• Community-based health services	• Community engagement and support	• AMR awareness at the community level
	• Objective: To develop community-based TB prevention and treatment services	• Objective: Community mobilization and participation in HIV prevention and control	• Objective: To carry out community education and publicity activities
	• Influencing factors: Community resources, infrastructure, and local policies	• Influencing factors: Limited community resources and insufficient participation	• Impact factors: Low public awareness and participation in AMR
	• Implementation strategy: Community health center, mobile clinics, community volunteers	• Implementation strategy: Community organization and mobilization, community basic projects	• Implementation strategy: Monitoring of community engagement and education effectiveness
	• Accountability mechanism: Monitoring of community engagement and program coverage	• Accountability mechanisms: Assessing community engagement and responsiveness	• Accountability mechanism: Monitoring of community engagement and education effectiveness
Institutional level	• Health system strengthening	• Organizational and institutional responsibilities	• Antibiotic management in medical institutions
	• Objective: To establish and strengthen health systems to effectively address TB	• Objective: Development of institutional policies and procedures	• Objective: To develop and implement antibiotic stewardship policies
	• Influencing factors: Health policy, financial input, human resources	• Influencing factors: Insufficient coordination between institutions	• Influencing factors: Insufficient antibiotic management in medical institutions
	• Implementation strategy: Health facilities upgrading, personnel training, and service process optimization	• Implementation strategy: Cross-departmental cooperation, policy consistency	• Implementation strategy: Supervise and audit the antibiotic use in medical institutions
	• Accountability mechanism: Monitoring the policy implementation rate and service quality	• Accountability mechanism: Monitoring agency performance and compliance	• Accountability mechanism: Monitoring policy implementation and effectiveness
Societal level	• Policy development and implementation	• Policy development and implementation	• Policy and regulatory level
	• Objective: To develop and implement effective TB control policies	• Objective: To develop and implement HIV prevention and control policies	• Objective: To develop and implement policies and regulations related to AMR
	• Influencing factors: Government commitment, laws and regulations, and regulatory mechanism	• Factors: Difficulty of policy implementation, uneven distribution of resources	• Factors: Lack of policies and regulations for AMR
	• Implementation strategy: International cooperation projects, financial assistance, and technical exchanges	• Implementation strategy: International cooperation projects, financial assistance, and technical exchanges	• Implementation strategy: International cooperation projects, financial assistance, and technical exchanges
	• Accountability mechanism: Monitoring the effects of international cooperation and resource sharing	• Accountability mechanism: Monitoring the effects of international cooperation and resource sharing	• Accountability mechanism: Monitoring the effects of international cooperation and resource sharing

Table 7 provides a comparative analysis of Stop TB, UNAIDS, and UNEP in their respective TB, AIDS, and antimicrobial resistance (AMR) action plans. The socio-ecological model-level comparative analysis summarized in Table 8 reveals commonalities and differences in governance and accountability for tuberculosis, HIV/AIDS, and microbial resistance (AMR). At the individual level, all three emphasized the importance of raising awareness, but each focused on specific implementation strategies. TB focused on health education, HIV focused on individual behavior change, and AMR focused on improving the knowledge of healthcare professionals and patients. At the interpersonal and community levels, all three focus on community engagement and support, as well as the strengthening of social networks. However, TB and HIV are more focused on community mobilization and communication between partners, while AMR emphasizes healthcare teams and doctor-patient communication. At the institutional level, all three action plans emphasize strengthening health systems and improving the quality of services, but AMR pays special attention to the development and implementation of antibiotic management policies. At the societal level, developing and implementing effective policies is a common goal, but TB and HIV are more focused on policy advocacy and funding allocation, while AMR focuses on policy and regulation development and the regulation of environmental emissions. Besides, all three stressed the importance of international cooperation, but AMR specifically mentioned the need to strengthen the involvement of the environmental sector and tackle emerging health threats with a One Health approach. Overall, while each disease has specific action plans and accountability mechanisms at different levels of the socio-ecological model, they all reflect the importance of cross-sectoral collaboration, community engagement, policy support and international cooperation in addressing public health challenges.

#### Integrated service for TB, HIV, and AMR

Funding for HIV/TB/AMR integration typically constitutes a subset of dedicated HIV, TB, or AMR budgets. For instance, major donors such as the Global Fund allocate substantial resources toward combating drug-resistant TB (DR-TB), exceeding US$2 billion during the 2020–2022 funding cycle for multidrug-resistant TB (MDR-TB) diagnosis and treatment within TB grants—directly contributing to AMR objectives. While the WHO End TB Strategy projected a requirement of approximately US$13 billion annually by 2022 for comprehensive TB programs, including MDR-TB, the actual available funding (only US$5–6 billion) signifies a critical underfunding gap for MDR-TB [61].

The AMR MPTFs country grants generally incorporate a TB component, such as strengthening TB laboratories for AMR surveillance. Consequently, a portion of this fund (projected to exceed US$70 million by 2024) supports TB-related AMR activities. Furthermore, research and development (R&D) funding for TB—encompassing novel antibiotics like bedaquiline and new vaccines—constitutes an integral component of the broader AMR investment landscape. Collectively, while TB represents a paramount priority within AMR strategic plans, dedicated funding remains inadequate, relying predominantly on general TB allocations supplemented by smaller AMR-specific grants.

Integrated HIV/TB projects have been instrumental in altering the trajectory of the TB/HIV co-epidemic. TB-associated mortality among people living with HIV (PLHIV) has declined significantly with the scale-up of integrated services. In 2023, 161,000 HIV-positive individuals died from TB, a substantial reduction from > over 400,000 annual deaths recorded in the mid-2000s [62]. This decline is largely attributable to the widespread implementation of antiretroviral therapy (ART) and isoniazid preventive therapy (IPT) within HIV care, coupled with routine HIV testing in TB care settings. Integration has yielded high service coverage: by 2018, 87% of TB patients in Africa knew their HIV status, and 86% of HIV-positive TB cases were receiving ART—a marked improvement from the approximately 55% testing coverage observed in 2015 [63, 64]. This linkage has demonstrably saved lives by ensuring timely treatment for both conditions in co-infected individuals. Global TB treatment success rates have consistently remained between 85 and 88% [61], indicating that integrating care for HIV-positive patients does not compromise treatment outcomes; rather, it enhances outcomes for co-infected individuals. Several countries (e.g., Eswatini, Rwanda) achieved significant reductions in AIDS-related mortality through the aggressive integration of TB/HIV services.

The integration of AMR-specific interventions into HIV and TB programs is comparatively recent; consequently, outcomes are primarily tracked in terms of systems enhancement and intermediate indicators. Key achievements include: the establishment of integrated AMR surveillance systems (encompassing HIV drug resistance, TB drug resistance, etc.), the development of One Health governance frameworks, and improved infection prevention and control (IPC) measures in healthcare facilities [65, 66]. By 2021, eight countries were actively implementing multisectoral AMR action plans supported by the AMR MPTF, thereby augmenting national capacities for detecting and responding to resistance threats [66].

TB/AMR integration focuses substantially on averting scenarios involving extreme economic burden. Treating a single MDR-TB patient can incur costs 10–20 times higher than treating a drug-susceptible TB case (exceeding US$10,000 per MDR-TB patient versus several hundred dollars for drug-susceptible TB in certain settings) [67]. Extensively drug-resistant TB (XDR-TB) entails even greater costs and is frequently associated with poor clinical outcomes. Therefore, preventing each case of MDR-TB—through effective initial treatment regimens and robust antimicrobial stewardship—represents significant cost savings. Integration facilitates prevention by rigorously applying directly observed therapy (DOT) to minimize acquired resistance and utilizing rapid diagnostics to expedite initiation of effective treatment. Moreover, integrating TB surveillance into broader AMR surveillance frameworks enhances cost-efficiency: infrastructure established to detect resistant TB can often be leveraged to identify other resistant infections, and vice versa. For example, integrated surveillance platforms (such as WHOs Tuberculosis Information System for Surveillance and Analysis, TISSA) capture TB drug resistance data alongside other AMR indicators [68]. To delineate distinctions between standalone disease programs and integrated initiatives, we summarizes key attributes of HIV, tuberculosis (TB), and antimicrobial resistance (AMR) initiatives from 2015 to 2024,details could be seen in Appendix Table 2 [64, 69–72]. Data and reports pertaining to HIV, TB, and AMR from the World Health Organization (WHO), the Global Fund to Fight AIDS, Tuberculosis and Malaria (Global Fund), the Joint United Nations Programme on HIV/AIDS (UNAIDS), and the AMR Multi-Partner Trust Fund (AMR MPTF) are prioritized.

A consistent finding across studies is that integrated approaches yield superior outcomes compared to disease-specific programs in addressing HIV, tuberculosis (TB), and antimicrobial resistance (AMR), although certain implementation gaps persist [25, 73–75]. Comparative research conducted between 2015 and 2024 provides quantitative evidence substantiating the advantages of integrated healthcare strategies, with several countries demonstrating exemplary practices [73, 76, 77].

South Africa has emerged as a leader in implementing integrated HIV/TB care programs, which have demonstrated measurable reductions in co-infection rates [78–80]. These integrated programs deliver HIV and TB services concurrently by the same healthcare provider during a single clinical encounter [78]. A study in Durban, South Africa, documented the adoption of an integrated model at the primary healthcare level for HIV and TB service provision [78]. Furthermore, a quality improvement intervention in KwaZulu-Natal, South Africa, elucidated key lessons for enhancing integrated HIV-TB services [81]. The SUTHI trial in South Africa sought to reduce mortality through integrated TB and HIV services in rural primary healthcare clinics [75]. Research indicates that integrating TB management into HIV care significantly improves TB case detection and treatment initiation [79]. Mortality rates among patients with HIV and tuberculosis decreased following the implementation of integrated HIV-TB treatment [80]. However, operationalizing integrated services continues to present substantial challenges [82].

Uganda has also demonstrated success with TB-HIV integrated care. A long-term integrated TB-HIV care model at the Infectious Diseases Institute Clinic in Kampala, Uganda, evidenced sustained positive impacts on tuberculosis treatment outcomes [83]. Additionally, research in rural Ugandan health facilities established that TB/HIV integration enhances antiretroviral therapy (ART) initiation and TB treatment outcomes among TB/HIV-coinfected patients [84].

In Zambia, integrating HIV care and treatment into tuberculosis clinics in Lusaka improved TB/HIV care coordination and facilitated earlier ART initiation [74]. This integration specifically targeted diagnostic and treatment delays inherent to fragmented approaches [74]. An implementation research logic model underscores the critical elements of adaptability, design quality, and complexity in integrating HIV/NCD care in Lusaka [85].

India contends with a substantial burden of HIV and TB co-infection [86]. Integrated healthcare systems in India are conceptualized to address these concurrent epidemics, necessitating high-quality, community-based health systems [87]. Evidence suggests that integrating TB/HIV treatment improves service delivery and maximizes favorable treatment outcomes [88].

Massachusetts has integrated its public health response for HIV, viral hepatitis, sexually transmitted infections (STIs), and tuberculosis through policies mandating contracted organizations to submit specimens to the Massachusetts State Public Health Laboratory for testing [89].

Zimbabwe’s Population-based HIV Impact Assessment survey identified missed opportunities for TB diagnostic testing among people living with HIV (PLHIV), underscoring the imperative for enhanced service integration [90]. Among adult PLHIV engaged in HIV care, a significant proportion were not screened for TB during their most recent HIV care visit [90].

These diverse examples collectively illustrate that integrated approaches enhance health outcomes by effectively addressing co-infections and optimizing service delivery. Quality improvement interventions and the deployment of electronic medical records represent valuable strategies for strengthening the implementation of integrated care [81, 85]. While comprehensive cost-benefit analysis data of integration of TB,HIV and AMR is unavailable, we can infer that integrating TB, HIV, and AMR policies may lead to cost savings through shared infrastructure, reduced duplication of efforts, and improved health outcomes from joint implementation. Above analysis and case studies demonstrate that integrated service delivery can yield higher treatment success rates and more efficient use of resources.

## Discussion

### Degree of integration across the three health priorities

The data shows that the integration of TB, HIV, and AMR varies significantly across different documents. The counts of each keyword indicate that these global health issues are not being addressed with equal frequency within WHO’s policy framework. There is a moderate level of integration between TB and HIV, as seen in documents like EB146/10 with 89 mentions of TB and 10 of HIV. This suggests that these two diseases are often discussed in the same context, likely due to their shared risk factors and the co-morbidity of TB in HIV-positive individuals. The integration of AMR with TB and HIV appears to be lower. For example, in document A68/20, there are 7 mentions of TB, 4 of HIV, but a significantly higher count of 115 for AMR. In all the WHO resolutions and decisions for TB, One Health was only mentioned once in EB146/10. One Health was not mentioned in all WHO resolutions and decisions for HIV. This indicates that while AMR is a prominent topic, it is not as frequently linked with TB and HIV in these documents. The overall frequency of such co-occurrence is low, with many documents having zero instances. This suggests that while the issues are discussed, they are not often integrated into a unified discussion in the same policy text. A71 proposed strong and urgent action on tuberculosis should align with the global antimicrobial resistance agenda, and speed up universal coverage of tuberculosis care and prevention in the context of the global agendas on antimicrobial resistance, health security and sustainable development. However, a cross-cutting public health approach for tackling antimicrobial resistance among TB, HIV, and AMR still remained at the initiative stage rather than the implementation improvement stage in January 2024.

WHO, Stop TB, UNAIDS and UNEP have both common and different governance and accountability mechanisms for TB, HIV and antimicrobial resistance in their respective action programmes. Besides, the WHO and Stop TB, UNAIDS and UNEP have demonstrated varying degrees of integration room in their action plans, governance and accountability mechanisms for TB, HIV and AMR. While each organization emphasized the importance of political leadership, legal and policy reform, adequate resource support, community engagement, prevention and education, treatment and care outreach, monitoring and evaluation systems, research and innovation, and international cooperation, they all focused on specific implementation strategies without sufficiently considering policy integration between different diseases and organizations. In terms of action plans, all four organizations emphasized political leadership, legal and policy reforms, adequate funding and other similar things. However, in terms of governance and accountability mechanisms, while these organizations share similar principles, such as strategic leadership, partnership building, advocacy for public health activities, standard-setting, technical support and capacity building, global monitoring and reporting, and the importance of funding, each disease area has its own unique set of objectives and implementation priorities. For example, tuberculosis and HIV may focus more on the treatment and prevention of specific diseases, while antimicrobial resistance emphasizes cross-sectoral collaboration and the rational use of antibiotics. In addition, the focus of research and innovation, the way communities engage, and the specifics of how accountability is implemented vary from disease to disease. From an integration perspective, although there are commonalities among the three health issues, the current governance mechanisms have not yet fully leveraged their synergies. For example, there is a high rate of comorbidity between TB and HIV, so they are discussed more often in combination in policy texts. In contrast, antimicrobial resistance, while widely discussed, has been more weakly linked to tuberculosis and HIV. Future work should therefore focus on enhancing integration across disease domains with a view to optimizing resource use and increasing cost-effectiveness through synergistic strategies.

The analysis based on the Cortellis Regulatory Intelligence database (Clarivate) reveals distinct patterns in global policy responses to TB, HIV, and AMR from 2015 to 2024. HIV-related policy documents (spanning 38 jurisdictions) were heavily dominated by regulatory actions and clinical guidance, with 35% consisting of FDA supplemental new drug approvals (often updating antiretroviral indications) and 11% being associated press releases. Similarly, TB documents (covering 29 jurisdictions) featured regulatory actions like new drug approvals or orphan designations (17%) alongside national program guidelines. In stark contrast, AMR policy instruments (originating from only 13 jurisdictions, primarily high-income countries/regions) focused overwhelmingly on strategic coordination: 28% were multi-stakeholder meeting records (e.g., One Health AMR meetings), followed by policy reports and action plan announcements, while product approval documents were notably scarce. This indicates AMR efforts prioritize framework development over therapeutic regulation.Geographical scope further highlights fragmentation. While a core group (US, EU, India, China, South Korea, Kenya) addressed all three areas, most countries exhibited siloed engagement. Several nations (e.g., Russia, Greece, Philippines, Bulgaria) appeared only in TB datasets, others (e.g., Singapore, Chile, Switzerland, Australia) solely in HIV datasets, and AMR activity was almost exclusively confined to high-income settings (e.g., Ireland, Sweden, Germany). Consequently, integrated policy attention across TB, HIV, and AMR remains limited to few jurisdictions, with HIV receiving the most policy focus and AMR the least. These findings underscore the absence of policy integration in global outputs and reinforce the critical need for broader adoption of unified frameworks across more countries.

### Cost-benefit for integration across the three health priorities

A significant lacuna in the argument for health service integration is the absence of robust quantitative evidence regarding its economic benefits. While vertical, disease-specific programs have demonstrated considerable success, they may engender inefficiencies, duplication of effort, and increased administrative burden. Conversely, an integrated approach promises substantial cost savings and efficiency gains. Empirical evidence from diverse settings supports the cost-effectiveness of integrated health service delivery.

For TB/HIV co-infection, service integration has proven to be a cost-effective intervention. A decision analysis study conducted in Brazil found that training healthcare workers to implement integrated tuberculin skin test (TST) screening and isoniazid preventive therapy (IPT) for HIV-positive patients was highly cost-effective. This strategy yielded an incremental cost-effectiveness ratio (ICER) of $2,273 per disability-adjusted life year (DALY) averted, substantially below the country’s per-capita GDP [91]. Another modeling study evaluating the “Three I’s” (Intensified case finding, Isoniazid preventive therapy, and Infection control) for HIV/TB concluded that strategies combining expanded antiretroviral therapy (ART) coverage with infection control and long-term IPT ranked among the most cost-effective approaches for preventing TB in high-burden settings [92]. These studies demonstrate that shared infrastructure, personnel, and patient pathways can reduce costs while simultaneously improving outcomes, such as ART uptake and TB treatment success rates [93].

Similarly, economic evaluations of antimicrobial stewardship (AMS) programs—a critical component of the antimicrobial resistance (AMR) response—consistently demonstrate a positive return on investment. A systematic review of hospital-based AMS programs found they were associated with significant reductions in antimicrobial consumption (19.1%), length of hospital stay (8.9%), and antimicrobial expenditure (33.9%) [94]. Another review noted that the average cost saving per patient attributable to AMS programs in the US was $732, primarily driven by reduced hospital stays [95]. Integrating AMR surveillance and stewardship activities into existing TB and HIV laboratory and clinical platforms could leverage these efficiencies, generating shared benefits. For instance, laboratory systems established for TB control can be expanded to accommodate broader AMR surveillance functions, optimizing resource utilization and accelerating rational antimicrobial use [96]. Although a comprehensive cost-benefit analysis of tripartite (TB/HIV/AMR) integration presents methodological complexity, the available evidence strongly suggests that synergistic programming can yield significant efficiency gains, reduce administrative burdens, and improve health outcomes. This constitutes a robust economic rationale for dismantling programmatic silos.

### Barriers to effective integration

Despite the clear rationale for integrating TB, HIV, and AMR services, multifaceted barriers exist beyond simple resource scarcity. A Social-Ecological Lens reveals these obstacles operating at distinct levels:

1. Individual Level:Healthcare worker shortages and insufficient training to manage co-morbidities comprehensively create a critical barrier. Under-staffing and increased workload, especially when integration occurs without added human resources, are major concerns for frontline providers [22, 26].
2. Interpersonal Level:Dual stigma associated with TB and HIV can deter patients from seeking care at integrated “1-stop-shop” facilities due to fear of identification [23]. Patient and provider attitudes, along with a lack of awareness about integration benefits, can generate resistance to new care models.
3. Institutiona Level:Persistent institutional silos, reinforced by disease-specific global initiatives and funding, create separate governance structures, reporting lines, and work cultures. This fragmentation hinders coordination and fosters competition over collaboration [22]. Weak infrastructure, including inadequate laboratory capacity and poor coordination between programs and administration, poses operational challenges [22, 26]. Technical hurdles include a lack of integrated clinical guidelines, differing diagnostic/treatment protocols, and incompatible data management systems [26]. Ensuring a consistent supply chain for drugs and diagnostics across three areas presents significant logistical hurdles.
4. Community Level:Community awareness and demand for integrated, people-centered care need cultivation to overcome stigma and build acceptance. Engaging communities is crucial to create this demand.
5. Societal Level:Competing funding mechanisms and donor preferences tied to single-disease mandates or performance indicators reinforce vertical programming, making it difficult to pool resources or report integrated outcomes [97]. Insufficient or unpredictable funding for overall health systems and specifically for NCDs/AMR can render integration an unfunded mandate, straining limited budgets [98]. The political economy, where departments or agencies are unwilling to cede control or resources, affects coordination.

Addressing these barriers demands a strategic approach harmonizing donor practices, strengthening health systems holistically, investing in workforce training, developing integrated data systems, and engaging communities.

### Identified areas for an integrated action

Qualitative analysis of World Health Assembly resolutions and EB decisions identified key areas for an integrated action across TB, HIV and antimicrobial resistance, including the continuum of services, surveillance, research and innovation, and strengthening health systems. These areas are potential entry points for incorporation into integrated global health strategies. The above results highlight the importance of monitoring, including the establishment of joint surveillance systems, coordination of indicators and reporting requirements, and sharing of best practices. This provides a comprehensive understanding of health challenges, to inform decision making and track progress. Research and innovation are also key areas, requiring interdisciplinary research to develop new tools and approaches with a more integrated approach to fundraising, research and resource sharing. This includes basic science research, clinical research, and business research to accelerate solution development to meet the needs of affected populations through a more collaborative resource mobilization and allocation among these diseases.

The above-mentioned areas provide a starting point for integrated action, the present policies lacked specific guidance for integration among the three diseases, though. integration is often seen as an additional or optional activity rather than a core component. However, to reach the full potential of integration, it is desirable for WHO to make more specific, actionable, and measurable integration solutions based on the social-ecological model.

Moreover, the siloed nature of global health funding mechanisms poses a significant obstacle to integrated action. Most donor funding and national budgets are earmarked for single diseases, creating a disincentive for health systems to invest in cross-cutting services—such as combined TB-HIV-AMR screening or shared laboratory infrastructure—even when these approaches are cost-effective. This fragmentation not only limits the scalability of integrated models but also perpetuates inequities, as resource-constrained settings lack the flexibility to reallocate funds toward holistic care. For example, a country with separate TB and HIV programs may prioritize disease-specific targets over coordinating treatment for co-infected patients, leading to suboptimal outcomes for those with complex needs.

Another critical barrier lies in the lack of intersectoral coordination at both national and subnational levels. While WHO resolutions emphasize integration, many health ministries continue to operate through disease-specific departments with limited communication channels, resulting in duplicated efforts and missed opportunities for synergy. This is compounded by the absence of standardized training for healthcare workers on integrated care, leaving frontline staff ill-equipped to manage co-morbidities or navigate overlapping guidelines. Patients, too, face challenges in accessing integrated services: stigma, geographic barriers, and inconsistent referral systems often force them to navigate multiple care pathways, increasing the risk of dropout and poor adherence.

To address these barriers, the social-ecological model offers a holistic framework that considers interactions across individual, community, and structural levels. At the individual level, targeted education campaigns could improve patient awareness of co-infection risks and the benefits of integrated care, while community-level interventions—such as mobile clinics or peer support groups—could enhance access for marginalized populations. At the structural level, WHO could play a pivotal role in harmonizing disease-specific policies, developing shared monitoring indicators, and advocating for flexible funding mechanisms that prioritize integration. For instance, a WHO-led framework could guide countries to align their TB, HIV, and AMR strategies under a single national health plan, with clear accountability mechanisms for progress.

Without addressing these systemic barriers—from funding silos to coordination gaps—integration will remain a theoretical goal rather than a practical reality. The urgency of TB, HIV, and AMR as interconnected public health threats demands a shift from disease-centric to people-centric approaches, where integration is not just encouraged but embedded into the core of global health governance.

### WHO needs better mechanisms to coordinate and allocate relevant resources based on the social ecological model

There is a high degree of integration between TB and HIV, with good links and long integration efforts between the two. WHO recommends a 12-component approach of collaborative TB-HIV activities, including actions for prevention and treatment of infection and disease, to reduce deaths [99]. However, the emerging status and challenge of AMR as a global health priority is less frequently integrated with the former 2. TB, HIV and AMR have some degree of integration in governance and accountability mechanisms, but there is still room for improvement. The specificity and operability of the integration of the three disease prevention and control resources depends on the political will, resources and capabilities of the Member States and other stakeholders. Although the World Health Organization recognizes the benefits of integration and advocates for integration in some areas, policy documents often deal in isolation. The lack of integration of WHO governance mechanisms, politicization of health issues and inadequate funding affect the effectiveness and sustainability of WHO responses to related health challenges, potentially leading to repeated efforts, fragmentation of services, and inefficient resource use.

**Fig. 4:**
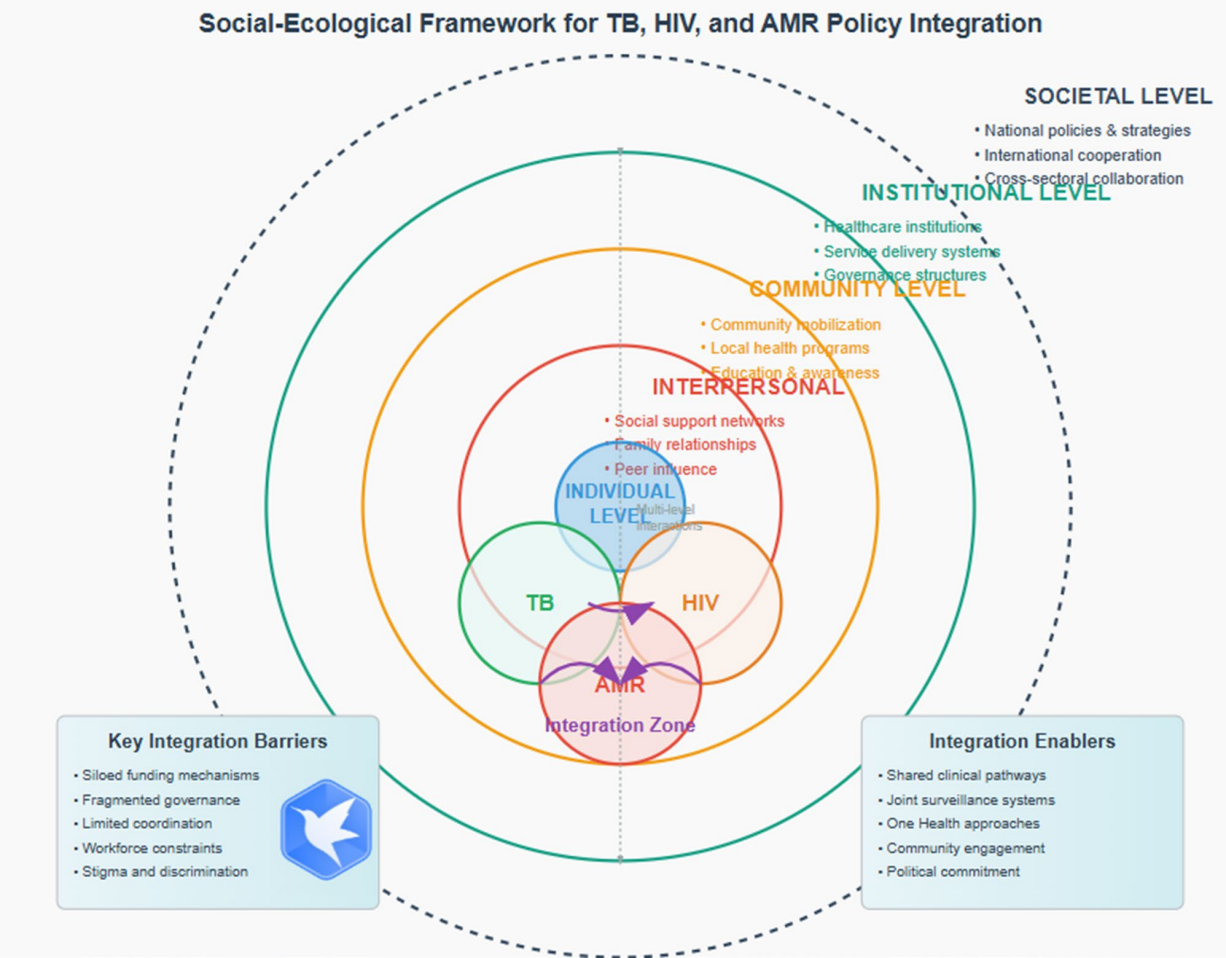
Social ecological framework for TB, HIV, and AMR integration

Therefore, WHO needs to design and implement an integrated solution based on the social ecological model. At the individual level, public awareness of these diseases can be raised through more comprehensive education and training,details presented in Fig. 4. At the interpersonal and community levels, disease management and social support can be enhanced by strengthening community engagement and support networks with a more integrated way. At the institutional level, medical institutions can provide more coordinated services through interdisciplinary collaboration. At the societal level, transnational health challenges can be addressed more effectively through more integrated international cooperation and policy coordination. At the service level, further integration and optimal allocation of resources can improve service efficiency and reduce fragmentation of the health system. These integrated efforts are expected to enhance the effectiveness of disease prevention and control measures and improve the efficiency and effectiveness of the use of human and financial resources in the context of resource constraints. A conceptual framework for integrated policy evaluation and implementation addressing tuberculosis, HIV, and antimicrobial resistance, grounded in the Social-Ecological Model, is presented in Appendix Table 3.

### Implications for national and global stakeholders

Our analysis has important implications for stakeholders involved in TB, HIV, and AMR responses – including governments, multilateral organizations (like WHO and partners), donors, civil society, and the private sector. WHO has set broad strategic frameworks (e.g., the End TB Strategy, Global Health Sector Strategy for HIV, Global Action Plan on AMR), whereas initiatives like Stop TB, UNAIDS, and UNEP focus on disease-specific or thematic action plans. WHO has advised Member States to implement integrated antimicrobial resistance surveillance and containment for HIV, TB, and malaria in national programs [100]. New techniques and methods are being recommended to support integration, such as greater use of whole-genome sequencing for surveillance and diagnostic network optimization (DNO) to improve lab networks for TB, HIV, and AMR [101, 102]. The adoption of national AMR strategies that explicitly include HIV and TB is a promising first step [103]. Ensuring regular access to effective treatments is also highlighted to reduce the emergence of drug resistance in HIV, TB, and malaria [104]. A One Health economic framework to estimate the cost of AMR has been suggested as a tool to evaluate the impact of AMR and justify investments [105]. However, similar integrated tools and frameworks need further development to truly promote integration of TB, HIV, and AMR in practice.

The thematic convergence indicates that integration is not merely aspirational but operationally feasible. Priority actions encompass: (i) joint national planning development and adoption of a shared core indicator set across tuberculosis (TB), human immunodeficiency virus (HIV), and antimicrobial resistance (AMR); (ii) establishment of integrated surveillance and laboratory networks—including stewardship and infection prevention platforms—under One Health governance frameworks; (iii) alignment of procurement systems and financing mechanisms to reduce duplication and enhance value for money; and (iv) implementation of community-centered delivery models and rights-based approaches to bolster demand generation, treatment adherence, and accountability across disease conditions. Potential trade-offs—such as coordination costs and risks of diluting disease-specific expertise—can be mitigated through phased integration, ring-fenced technical capacity, and explicit accountability structures.

To translate integration into practice, stakeholders must adopt a social–ecological framework—simultaneously addressing individual behaviors (e.g., patient adherence and stigma reduction), interpersonal dynamics (including family and community support systems), institutional practices (e.g., service integration within clinical facilities), and societal factors (notably policy formulation and funding allocation). A policy architecture grounded in the social–ecological model merits promotion for analyzing and guiding integrated policy development across all governance levels. While TB, HIV, and AMR action plans maintain distinct objectives at various tiers, they collectively underscore the critical importance of cross-sectoral collaboration, sustained community engagement, enabling policy environments, and international cooperation in confronting public health challenges. Effective cross-domain integration enhances programmatic efficacy, optimizes resource utilization, and amplifies synergies between disease-specific initiatives. By consolidating action plans across diverse stakeholders and disease areas, global health actors can more effectively address these interconnected threats and advance progress toward the Sustainable Development Goals (SDGs).

### Limitations

While the study provides valuable insights for incorporating TB, HIV, and AMR into the WHO action plans, there are limitations to be considered.

Limitations include: focusing only on specific policy documents may not fully reflect the relevant activities and initiatives inside and outside WHO; the comparison mainly relies on qualitative methods and may be influenced by investigator bias; the study did not include primary informant data and lacked stakeholder perspectives and experiences; by focusing only on specific health priorities, the results may not be generalizable to other areas; the impact and effectiveness of integrated action plans at the national level need to be thoroughly evaluated to determine their success in addressing complex issues and achieving sustainable outcomes; word frequency analysis may identify documents mentioning multiple diseases without demonstrating proper policy integration. This evaluation should consider factors such as stakeholder engagement, resource allocation, monitoring and evaluation mechanisms, and the overall coordination of efforts across different sectors. And more evidence directly linking policy-level integration with service delivery integration need to be explored. Only by understanding the strengths and weaknesses of these plans can policymakers make informed decisions on how to improve and optimize their implementation for maximum impact.

Despite its limitations, the study is expected to make a valuable contribution to the formulation of a global strategy to address TB, HIV and AMR in a holistic way. Future studies could explore a wider range of documents and data sources, incorporate major data collection methods, and explore other health priorities and areas of work. Action plan impact and effectiveness can be assessed through case studies or by evaluating implementation efforts at the national level. Ultimately, addressing limitations and outcomes requires a collaborative and multidisciplinary approach to build a more equitable, resilient and sustainable global health system.

## Conclusion

Action plans for tuberculosis, HIV/AIDS, and microbial resistance have demonstrated a degree of integration within the framework of socio-ecological models. However, the integration effect of these three diseases has great room for improvement based on socio-ecological models. The study suggests a multifaceted and multi-level approach to address the integration of TB, HIV, and AMR. The approach should be based on an integration solution using the social ecological model and principles of person-centered care and participation of multiple stakeholders to meet the needs of different Member States. The WHO needs to play a key role in driving transformation and promoting the integrated application of the social ecological model in more areas to better address global health challenges in a resource-constrained environment. Not only patients need integrated health services, WHO also need an integrated solution to solve the challenges faced by tuberculosis, HIV/AIDS, and microbial resistance, while harmonizing with Stop TB, UNAIDS, and UNEP. Despite disease-specific plans and accountability mechanisms, TB, HIV, and AMR policies converge on shared enablers—cross-sectoral collaboration, community engagement, supportive policy/legislation, and international cooperation. Elevating these commonalities from commitments to operational design provides a realistic pathway to integrated governance and delivery. In resource-constrained settings, such integration is both feasible and necessary to accelerate progress toward SDG targets while improving efficiency and equity.

Social-Ecological model is a useful tool to develop integration strategies between the three diseases.The World Health Organization (WHO) should devise an integrated policy framework, grounded in a social ecological model, to offer clear definitions, standards, and guidance for a holistic approach to disease management. Concurrently, a comprehensive governance and coordination mechanism should be set up across horizontal and vertical dimensions, encompassing individual, interpersonal, community, institutional, and societal levels, to foster interdepartmental collaboration and integrated solutions and strategies. The WHO should assist countries in adopting integrated methodologies, establishing standardized indicators, and developing monitoring and evaluation frameworks, as well as in training staff and partners. Governments are tasked with formulating integrated national plans and policies to reinforce health systems and infrastructure and to encourage community engagement. Donors and funding agencies should back integrated approaches and prioritize investments in interdisciplinary projects. The private sector, civil society, and affected communities should also be involved in the design of integrated responses.

The integration of TB, HIV, and AMR into global health strategies necessitates a fundamental shift from isolated and biomedical paradigms to comprehensive and social ecological approaches. The findings offer a roadmap for enhancing the integration of TB, HIV, and AMR into the WHO action plan and for establishing more collaborative and effective global health systems.

The COVID-19 pandemic highlighted the urgent need for a global framework that enhances surveillance and promotes a more integrated, holistic health system. Over the years, WHO has steadily developed and expanded its One Health programs to address emerging global health threats such as zoonotic diseases, antimicrobial resistance (AMR), and environmental risks. At the same time, WHO is also facing a complex set of challenges as it addresses the health impacts of climate change, especially amid limited global health funding and resource constraints. For WHO to maximize its efficiency and impact, it should adopt specific, actionable, and measurable integration implementation strategies. A paradigm shift is also needed for national and global stakeholders to view integration as essential, not optional. Integration is a long-term endeavor that demands political commitment, leadership, and investment. It challenges the status quo and necessitates the adoption of new working methods. Consequently, it is imperative to implement more efficient and effective integrated practices, grounded in a social ecological model.

## Electronic supplementary material

Below is the link to the electronic supplementary material.

Supplementary Material 1

## Data Availability

All data relevant to the study are included in the article and relevant documents could be acquired from the website of WHO, Stop TB, UNAIDS, UNEP and Cortellis Regulatory Intelligence database.

## Abbreviations

WHO: World Health Organization
WHA: World Health Assembly
EB: the Executive Board
UNAIDS: Joint United Nations Programme on HIV and AIDS
UNEP: United Nations Environment Programme
Stop TB: Stop TB Partnership
Gavi: The Global Alliance for Vaccines and Immunizations
TB: Tuberculosis
MDR-TB: Multi-drug-resistant Tuberculosis
HIV: Human Immunodeficiency Virus
AIDS: Acquired Immune Deficiency Syndrome
HIVDR: HIV Drug Resistance
AMR: Antimicrobial Resistance
TrACSS: tracking Antimicrobial Resistance Self-Assessment Survey
NCDs: Noncommunicable Diseases
SDGs: Sustainable Development Goals
