# Supplementary material for: Evaluation of integration in WHO’s tuberculosis, HIV, and antimicrobial resistance policies through the social-ecological lens
Source: Global Health. 2025 Sep 29;21:53. doi: 10.1186/s12992-025-01150-3 (PMC12482740; doi:10.1186/s12992-025-01150-3)
Supplement: Supplementary file 1 — Supplementary Material 1 [file 12992_2025_1150_MOESM1_ESM.docx]

## Appendix

The table below lists the 75 countries/regions included in this study from the Cortellis regulatory intelligence database(Clarivate) for quantitative trend analysis ， along with their income group classification according to the World Bank for fiscal year 2024.

## Table1 List of 75 Sample Countries or Districts and Income Classification

| Country/Region | ISOCode | ncomeGroup | Country/Region | ISO  Code | incomeGroup |
| --- | --- | --- | --- | --- | --- |
| Argentina | ARG | Upper-middle | Australia | AUS | High |
| Austria | AUT | High | Belgium | BEL | High |
| Brazil | BRA | Upper-middle | Canada | CAN | High |
| Switzerland | CHE | High | Chile | CHL | High |
| China | CHN | Upper-middle | Colombia | COL | Upper-middle |
| Germany | DEU | High | Denmark | DNK | High |
| Egypt， Arab Rep. | EGY | Lower-middle | Spain | ESP | High |
| Finland | FIN | High | France | FRA | High |
| United Kingdom | GBR | High | Greece | GRC | High |
| Indonesia | IDN | Lower-middle | India | IND | Lower-middle |
| Ireland | IRL | High | Israel | ISR | High |
| Italy | ITA | High | Japan | JPN | High |

| Country/Region | ISOCode | lncomeGroup | Country/Region | ISOCode | IncomeGroup |
| --- | --- | --- | --- | --- | --- |
| Kenya | KEN | Lower-middle | Korea， Rep. | KOR | High |
| Mexico | MEX | Upper-middle | Malaysia | MYS | Upper-  middle |
| Nigeria | NGA | Lower-middle | Netherlands | NLD | High |
| Norway | NOR | High | New Zealand | NZL | High |
| Pakistan | PAK | Lower-middle | Philippines | PHL | Lower-  middle |
| Poland | POL | High | Portugal | PRT | High |
| RussianFederation | RUS | Upper-middle | Saudi Arabia | SAU | High |
| Singapore | SGP | High | Sweden | SWE | High |
| Thailand | THA | Upper-middle | Turkey | TUR | Upper-  middle |
| South Africa | ZAF | Upper-middle | United States | USA | High |

Table 2 Standalone vs. Integrated Approaches among TB, HIV, and AMR

|  | **Standalone Programs** | **Integrated Projects** |
| --- | --- | --- |
| Funding | HIV: Approximately US$19.8 billion was available for HIV programs in low- and middle-income countries (LMICs) in 2023. This falls nearly US$9.5 billion short of the annual funding requirement projected by 2025 to meet UNAIDS targets [69]. The Global Fund has contributed approximately 28% of international HIV financing, having invested US$26.6 billion in HIV programs since 2002 [70].  TB:Global tuberculosis (TB) funding has consistently lagged behind established targets. In 2023, only US$5.7 billion was available for TB prevention and care worldwide, representing approximately 26% of the annual target of US$22 billion set by the United Nations for achievement by 2027 [71]. The Global Fund serves as the largest external funder for TB, providing approximately 76% of international TB financing. Since its inception, it has approved over US$11 billion for TB programs [62], including US$9.9 billion in standalone TB grants as of 2024 [70]. Domestic funding remains critical, exemplified by high-burden countries such as China, which largely self-finance their TB programs.  AMR: Dedicated funding for antimicrobial resistance (AMR) emerged significantly in the late 2010s. The flagship initiative is the AMR Multi-Partner Trust Fund (MPTF), established in 2019 by the WHO, FAO, WOAH (World Organisation for Animal Health), and UNEP. It receives support from multiple donors, including Germany, the Netherlands, Sweden, and the UK Fleming Fund [65]. By 2021, the MPTF was financing AMR action plans in 8 countries alongside global projects [66]. Although the fund's initial capitalization was modest (tens of millions of US dollars), its objective is to catalyze national investments. The Global Action Plan on AMR (2015) stimulated donor commitments, for example, the Fleming Fund (~£265 million for laboratory strengthening), US CDC and USAID AMR programs, and EU Horizon funding for AMR research and development. Estimating annual global AMR financing is complex due to its distribution across human health, animal health, and environmental sectors. | The Global Fund and its partners allocate substantial resources to collaborative HIV/TB initiatives, exemplified by a cumulative investment of US$5.5 billion in HIV/TB programs as of June 2024 [70]. These resources facilitate integrated service delivery for patients with co-infection. National governments and international donors prioritize high-burden countries for HIV/TB program integration.  In contrast, integration of HIV and antimicrobial resistance (AMR) initiatives remains nascent. Funding typically originates from broader health security or dedicated AMR initiatives instead of core HIV budgets. For instance, the AMR Multi-Partner Trust Fund (MPTF), established in 2019, supports One Health AMR activities across 10 countries [65]. Investments focus on antimicrobial stewardship within HIV care settings (e.g., appropriate antibiotic use for HIV-related opportunistic infections) and surveillance of HIV drug resistance. While constituting a minor fraction of overall HIV funding, these efforts are critical for sustaining long-term treatment efficacy [72]. AMR/HIV integration funding is not a distinct allocation stream; it is generally embedded within larger programmatic frameworks.  TB/HIV integration constitutes a priority for major funding entities. The Global Fund, for example, had invested US$1.9 billion in cross-cutting TB/HIV programs by 2024, supplementing disease-specific grants [70]. Integrated financing frequently manifests as designated portions within HIV grants utilized for TB activities, or conversely, TB grants supporting HIV interventions.  TB and AMR integration receives support under global AMR initiatives and targeted programs addressing multidrug-resistant tuberculosis (MDR-TB). The AMR MPTF (a multisectoral fund), for example, provides resources to strengthen integrated AMR surveillance, including monitoring resistance in *Mycobacterium tuberculosis* [65]. The Fleming Fund and other donors also invest in laboratory capacity for drug-resistant TB detection as part of broader AMR strengthening efforts. Within TB budgets globally, an increasing proportion is allocated to drug-resistant TB, encompassing MDR-TB and extensively drug-resistant TB (XDR-TB) treatment, scaling up rapid molecular diagnostics, and research and development (R&D) for novel therapeutics and vaccines. Notably, global TB R&D funding reached approximately US$1 billion in 2023, representing only 20% of the estimated US$5 billion annual requirement [61]. Integrating TB more explicitly into national AMR plans may unlock additional financing from health security sources, given tuberculosis's significant contribution to global AMR-associated mortality. |
| Countries Involved | HIV: Nearly all countries implement dedicated HIV programs due to the epidemic's global nature. Nevertheless, sub-Saharan Africa remains the epicenter, hosting the majority of the estimated 39.9 million people living with HIV in 2023 [70].  TB: Standalone TB programs operate globally, yet approximately 87% of incident TB cases occur within the 30 designated high-burden countries (HBCs) [61].  AMR: One Health-focused AMR programs are increasingly implemented worldwide. The AMR Multi-Partner Trust Fund (MPTF) initially supported 8 countries by 2021 and expanded to 10, including Kenya, Ethiopia, Ghana, Zimbabwe, Indonesia, and Peru [66]. Notably, several MPTF-supported countries bear significant HIV burdens (e.g., Kenya, Zimbabwe), presenting distinct opportunities to integrate AMR initiatives within HIV care frameworks [65]. | Thirty high HIV/TB burden countries, predominantly located in Africa, constitute the primary focus of integrated HIV/TB initiatives. In 2023, among 10.8 million new tuberculosis (TB) cases, approximately 662,000 occurred among people living with HIV (PLHIV), and 13% of TB-related fatalities were among HIV-positive individuals [62]. Consequently, integrated TB/HIV programs are prioritized in settings where HIV drives TB transmission dynamics. Within sub-Saharan Africa, TB/HIV co-infection is highly prevalent; notably, South Africa, Mozambique, Kenya, Tanzania, and 12 additional nations are designated by the World Health Organization (WHO) as "high TB/HIV burden" countries. In certain regions of these nations, over 50% of TB patients test positive for HIV. Recognizing TB as a principal antimicrobial resistance (AMR) threat, integrated AMR/TB efforts specifically target countries experiencing substantial burdens of multidrug-resistant TB (MDR-TB). The WHO identifies 30 high MDR-TB burden countries—including India, China, Russia, South Africa, and the Philippines—which collectively account for the majority of global MDR-TB cases [73]. The country list under the AMR Multi-Partner Trust Fund (AMR MPTF)—comprising Cambodia, Ethiopia, Kenya, Indonesia, among others—exhibits significant overlap with TB priority countries. For instance, Indonesia and Ethiopia, facing elevated rates of both TB and MDR-TB, are included in the AMR MPTF, ensuring TB is incorporated into their One Health AMR responses. Integration of AMR and HIV considerations tends to occur in countries with large HIV burdens where healthcare-associated infections and extensive antibiotic utilization within HIV care pose significant challenges. For example, sub-Saharan African countries with high HIV prevalence, such as South Africa, Kenya, and Uganda, are also contending with rising AMR (burdens, including resistant bacterial infections among PLHIV. |
| Implementers | HIV: National HIV/AIDS Control Programs (typically under Ministries of Health), international agencies (notably UNAIDS and WHO), and NGOs/community-based organizations are primarily responsible for implementing standalone HIV interventions. Major donors, (including the Global Fund and the President's Emergency Plan for AIDS Relief (PEPFAR), provide funding and coordination for these efforts.  TB: National TB Programs (NTPs) lead standalone tuberculosis control initiatives, frequently operationalized through networks of dedicated TB clinics and community health workers. The WHO Global TB Programme offers essential technical guidance, encompassing strategies such as Directly Observed Treatment, Short-course (DOTS) and standardized treatment guidelines. Non-governmental organizations (e.g., Stop TB Partnership, The International Union Against Tuberculosis and Lung Disease) and community volunteers (focused on patient support) additionally implement TB-related activities.  AMR: Virtually all member states endorsed the Global Action Plan on Antimicrobial Resistance (AMR) in 2015 and had developed National AMR Action Plans (NAPs) by 2024. However, only approximately 50 to 60 nations had progressed to active implementation phases with significant funding allocations. The Antimicrobial Resistance Multi-Partner Trust Fund (AMR MPTF) concentrates its support on 10 priority countries across Africa, Asia, and Latin America. | Integrated service delivery is facilitated by joint teams from national HIV and tuberculosis (TB) programs. Numerous countries have implemented a "one-stop shop" model, involving the co-location of HIV and TB clinics or the utilization of shared healthcare personnel for both diseases. The World Health Organization (WHO) and its partners support this integration through established guidelines[73]. The Global Fund frequently structures grants to encompass both HIV and TB components in settings with high co-infection prevalence. The Quadripartite (WHO, Food and Agriculture Organization [FAO], World Organisation for Animal Health [OIE], United Nations Environment Programme [UNEP]) coordinates the Antimicrobial Resistance Multi-Partner Trust Fund (AMR MPTF) projects, collaborating with ministries of health to incorporate HIV-related considerations within One Health plans[65].  Within integrated AMR/TB initiatives, **TB programs and AMR programs collaborate closely**. For instance, TB reference laboratories implement novel diagnostic technologies (e.g., line probe assays, Xpert MTB/RIF), developed primarily through TB funding streams, which concurrently advance AMR surveillance objectives. WHO's TB and AMR departments collaborate on guideline development for managing drug-resistant tuberculosis (DR-TB), thereby integrating core AMR principles into TB treatment standards. Furthermore, given the significance of TB within the AMR landscape, One Health AMR projects frequently include TB experts within their technical working groups.  At the national level, the National Tuberculosis Program (NTP) contributes data on TB drug resistance into the national AMR surveillance system. |
| Beneficiaries | HIV:Primarily people living with HIV (PLHIV) and populations at elevated risk of infection – which include key populations (e.g., sex workers, men who have sex with men [MSM], people who inject drugs) and adolescent girls and young women (AGYW). In 2023 alone, 17.9 million people received HIV prevention services, including 8 million individuals from key populations [70].  TB: Primarily individuals with active TB disease and their communities. Dedicated TB programs focus on identifying undetected TB cases (addressing the approximately 2.7 million case detection gap), ensuring successful treatment for diagnosed patients, and providing preventive therapy to high-risk groups. In 2023, 8.2 million new TB cases were diagnosed and notified [61]. Additional beneficiaries comprise household contacts (through systematic screening and preventive treatment provision) and the general population (via reduced TB transmission).  AMR:The entire population derives benefit from effective antimicrobial resistance (AMR) control, as it mitigates the emergence of untreatable infections. In the near term, patients requiring hospitalization or treatment for common infections derive direct benefit from preserved antibiotic efficacy. Specific patient groups experience significantly enhanced protection, including transplant recipients, individuals undergoing chemotherapy, persons living with HIV, and others with compromised immune systems, as they exhibit heightened vulnerability to drug-resistant infections. | Individuals experiencing co-infection or at elevated risk thereof. At a population level, containment efforts targeting HIV, tuberculosis (TB), and antimicrobial resistance (AMR) yield benefits for all patients and the general public. Within the context of HIV infection, people living with HIV (PLHIV) derive critical benefit from effective management of co-infections (such as TB and bacterial pneumonias) when antimicrobial agents retain their efficacy. PLHIV further benefit from surveillance systems that effectively constrain the emergence of antiretroviral (ARV) resistance, thereby preserving the potency of ARV regimens. In essence, integrated AMR initiatives safeguard HIV patients by maintaining the therapeutic utility of essential medications and mitigating the incidence of drug-resistant infections within clinical settings. Patients co-infected with TB and HIV receive seamless care; for instance, integrated service delivery enables concurrent administration of antiretroviral therapy (ART) and anti-TB regimens within a single facility, enhancing treatment adherence. Additionally, TB patients without HIV infection benefit from ascertaining their HIV status and accessing preventive interventions, while HIV-positive individuals benefit from TB prevention or early detection. The broader population benefits from reduced cross-infection: effective TB treatment among PLHIV diminishes community transmission of TB, and effective HIV treatment reduces the reservoir of immunocompromised individuals susceptible to propagating TB infection. Direct beneficiaries include patients with drug-resistant TB (DR-TB), who experience improved diagnostic capabilities and therapeutic outcomes under integrated approaches (e.g., accelerated identification of multidrug-resistant (MDR) strains, access to novel all-oral MDR-TB regimens demonstrating enhanced success rates of approximately 68%)[61]. Broader beneficiaries encompass communities experiencing reduced transmission of resistant TB strains. Integration of TB management within AMR stewardship programs confers advantages even to patients with drug-susceptible TB, as optimized management reduces the incidence of acquired resistance during therapy. Furthermore, healthcare workers benefit from standardized protocols and enhanced resources for infection control when TB is incorporated into AMR planning frameworks, thereby mitigating their occupational risk of DR-TB exposure. |

Table3 Framework for integrated policy evaluation and implementation of TB, HIV and AMR

| Levels | Dimensions | Matters of integration | Integrated evaluation indicator |
| --- | --- | --- | --- |
| Individual level | Know-how | - The level of public knowledge of TB, HIV and AMR - Knowledge of an integrated multi-disease prevention approach in high-risk groups | - Basic knowledge of the public about the three diseases and comprehensive prevention methods were assessed by questionnaire - Conduct special investigations for high-risk groups to understand their awareness of comprehensive prevention methods for multiple diseases |
|  | Behavior change | - Behavioral change to an integrated multi disease prevention approach for high-risk groups | - Assess actual behavior change in multi-disease prevention approaches in high risk groups through a behavior change program |
|  | Self-management | - Patient self-management capacity for multi-disease management | - Assessment of patient self-management in multi disease management through patient self-report and medical records |
| Interpersonal level | Social support | - Coverage of the community support network for patients with multiple diseases | - Assess whether the community support network covers both TB, HIV and AMR patients and provides integrated support services |
|  | Discrimination decreased | - Reduce discrimination against patients with multiple diseases | - Discrimination against patients with multiple diseases was assessed through questionnaires and community feedback |
|  | Communication skill | - Improve the communication skills between patients and healthcare professionals | - Assess patient communication skills with providers through training programs and patient feedback |
| Community level | Community participation | - Community members participate in joint multi-disease intervention activities | - Statistical participation rates in community health promotion activities that include the three disease interventions |
|  | Service coverage | - Accessibility of multi disease integrated services | - Assess the accessibility of integrated multi disease services such as the establishment of a one-stop service center |
|  | Educational activities | - Multi-disease joint health education activities | - Statistics the number and participation rate of multi-disease combined health education activities |
| Institutional level | Quality of service | - Quality of integrated services-disease services | - Assess the quality of multi disease integrated services through patient satisfaction surveys and quality assessment reports |
|  | Policy implementation | - Implementation of comprehensive multi-morbidity policies | - Assess the implementation of comprehensive multi disease policies, such as the implementation of policy documents |
|  | Interdisciplinary cooperation | - Number of multi-disease joint diagnosis and treatment projects among medical institutions | - Count the number of multi-disease joint diagnosis and treatment projects among medical institutions |
| Societal level | Policy integration | - Coordination and consistency of multi-disease policies | - Assess national policy coordination and consistency in the area of multiple diseases, such as whether joint multiple disease management is explicitly mentioned in the policy document |
|  | Funding | - Funding input multi-disease management | - Assess funding inputs for multi disease management, such as budget allocation and actual use |
|  | Monitoring and evaluation | - Surveillance and evaluation of multi-disease services | - Assess the monitoring and assessment of multi-disease integrated services, such as periodic monitoring and evaluation reports |
|  | Service integration | - Integration of multi-disease integrated services | - Assess the integration of multi-disease integrated services, such as the establishment and operation of one-stop service centers |
|  | Managerial hierarchy | - Governance structure for multi-disease management | - Assess governance structures for multi-disease management, such as the organizational structure for policy making and implementation |
|  | Accountability mechanism | - Accountability mechanisms for multi-disease management | - Accountability mechanisms for assessing multi-disease management, such as transparency and accountability for policy implementation and resource use |
